# PTEN status is a crucial determinant of the functional outcome of combined MEK and mTOR inhibition in cancer

**DOI:** 10.1038/srep43013

**Published:** 2017-02-21

**Authors:** Michele Milella, Italia Falcone, Fabiana Conciatori, Silvia Matteoni, Andrea Sacconi, Teresa De Luca, Chiara Bazzichetto, Vincenzo Corbo, Michele Simbolo, Isabella Sperduti, Antonina Benfante, Anais Del Curatolo, Ursula Cesta Incani, Federico Malusa, Adriana Eramo, Giovanni Sette, Aldo Scarpa, Marina Konopleva, Michael Andreeff, James Andrew McCubrey, Giovanni Blandino, Matilde Todaro, Giorgio Stassi, Ruggero De Maria, Francesco Cognetti, Donatella Del Bufalo, Ludovica Ciuffreda

**Affiliations:** 1 Medical Oncology 1, Regina Elena National Cancer Institute, Rome, Italy; 2 Translational Oncogenomic Unit, Regina Elena National Cancer Institute, Rome, Italy; 3 Experimental Chemotherapy Laboratory, Regina Elena National Cancer Institute, Rome, Italy; 4 ARC-Net Research Centre and Department of Pathology, University of Verona, Verona, Italy; 5 Biostatistics, Regina Elena National Cancer Institute, Rome, Italy; 6 DiBiMIS, University of Palermo, Palermo, Italy; 7 Data Analysis Unit, Siena Biotech S.p.A. Siena, Italy; 8 Department of Hematology, Oncology and Molecular Medicine, Istituto Superiore di Sanità, Rome, Italy; 9 Section of Molecular Hematology and Therapy, Department of Leukemia, The University of Texas MD Anderson Cancer Center, Houston (TX), USA; 10 Department of Microbiology & Immunology, Brody School of Medicine, East Carolina University, Greenville (NC), USA; 11 Scientific Direction, Regina Elena National Cancer Institute, Rome, Italy

## Abstract

Combined MAPK/PI3K pathway inhibition represents an attractive, albeit toxic, therapeutic strategy in oncology. Since PTEN lies at the intersection of these two pathways, we investigated whether PTEN status determines the functional response to combined pathway inhibition. PTEN (gene, mRNA, and protein) status was extensively characterized in a panel of cancer cell lines and combined MEK/mTOR inhibition displayed highly synergistic pharmacologic interactions almost exclusively in PTEN-loss models. Genetic manipulation of PTEN status confirmed a mechanistic role for PTEN in determining the functional outcome of combined pathway blockade. Proteomic analysis showed greater phosphoproteomic profile modification(s) in response to combined MEK/mTOR inhibition in PTEN-loss contexts and identified JAK1/STAT3 activation as a potential mediator of synergistic interactions. Overall, our results show that PTEN-loss is a crucial determinant of synergistic interactions between MAPK and PI3K pathway inhibitors, potentially exploitable for the selection of cancer patients at the highest chance of benefit from combined therapeutic strategies.

Cancer is increasingly recognized as a signaling disease. The RAF/MEK/ERK (MAPK) and PI3K/AKT/mTOR (PI3K) pathways cooperate to govern fundamental physiological processes, such as cell proliferation, differentiation, metabolism and survival^1^,^2^,^3^. Constitutive activation of one or both these pathways is a commonly occurring event and has been implicated in the initiation, progression and metastasis of solid and hematologic malignances^4^,^5^,^6^,^7^,^8^. Extensive cross-talk occurs between the MAPK and PI3K pathways, but their relationship is complex, so that pharmacologic interference at a single point of the network may actually result in the “paradoxical”, and often “undesired” from a therapeutic point of view, activation of the same or the alternative pathway, thereby leading to cancer cell survival and drug resistance^9^,^10^,^11^.

In this context, combined inhibition of both MAPK and PI3K is being tested as a potential strategy to overcome/delay resistance and widen the scope of sensitive cancer patients^4^,^9^,^10^,^12^,^13^,^14^,^15^,^16^. However, combined pathway inhibition in the clinical setting often requires substantial reductions of each single agent dose. Moreover, this type of strategy implies increased monetary and toxicity costs, which represent a high risk for both individual patients and the society as a whole, should it fail to demonstrate more than additive benefits. Thus, the identification of putative biomarkers of synergistic therapeutic interactions will be crucial to successfully develop combination strategies in the clinical setting, allowing for the selection/enrichment of patients who are most likely to benefit^12^,^17^,^18^.

Our group has recently reported on a novel crosstalk mechanism between the MAPK and PI3K pathways, whereby constitutive ERK activation represses PTEN expression in melanoma and other cancer models. These findings bear important functional consequences, since in cellular contexts in which PTEN is unaltered MEK blockade leads to increased PTEN protein expression, which plays an important, albeit not exclusive, role in the antitumor and anti-angiogenic activities of MEK inhibitors^9^,^10^.

Based on this rationale, we evaluated the role PTEN status has in modulating the growth inhibitory activity of single or combined MEK and mTOR inhibition. Our results show that growth inhibitory synergism with combined MAPK/PI3K inhibition is almost invariably observed in cells with PTEN-loss, but not in tumor cells with an intact PTEN. PTEN expression or lack thereof causally modifies both signaling perturbations and functional responses induced by combined MEK and mTOR inhibition, suggesting that PTEN-loss maybe proposed as a potential selection/stratification factor for clinical trials employing such combinations.

## Results

### PTEN profiling in human cancer cell lines

**Figure 1:**
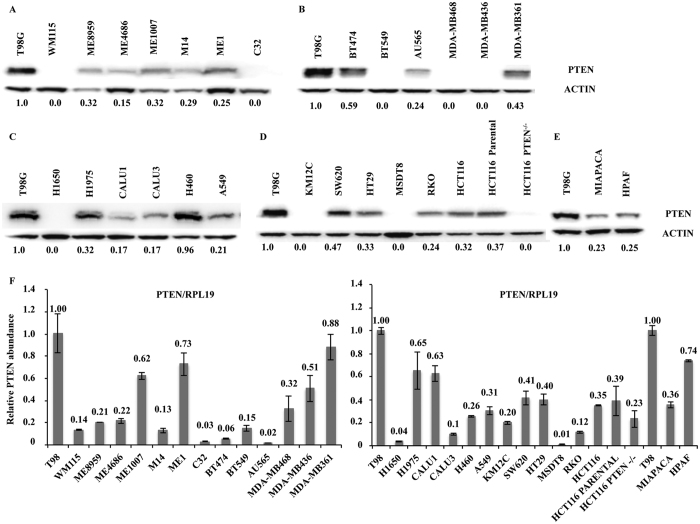
PTEN expression in different cancer cell lines. (**A**–**E**) The cells, divided according to histological origin, were lysed and analyzed by Western Blotting using antibodies specific for PTEN. Western blot with antibodies specific for β-actin are shown as protein loading and blotting control. The T98G cells were used as a positive control for PTEN expression. (**F**) The presence of PTEN was detected by real-time PCR in all cell lines analyzed previously. Results were evaluated as ΔΔct of PTEN tested relative to RPL19 and expressed as the ratio assuming the levels in T98G positive control cells as 1.0.

To investigate the role of PTEN in modulating the response to MAPK or PI3K pathway inhibition, panels of thirty tumor cell lines of different histological origin (melanoma, n = 7; breast cancer, n = 6; non-small cell lung cancer, n = 6; colorectal cancer, n = 8; pancreatic adenocarcinoma, n = 2; glioblastoma, n = 1; Table 1) were analyzed for PTEN gene status. To this purpose, DNA extracted from each sample was amplified by multiplex PCR for the PTEN gene and an adequate library for deep sequencing was obtained. The mean read length was 101 base pairs and a mean coverage of 1823× was achieved, with 94.5% target bases covered at more than 100×. A minimum coverage of 50× was obtained in all cases. Results are summarized in Table 1. PTEN expression was further analyzed at the mRNA and protein levels by RT-qPCR and Western blotting in all cell lines. As shown in Fig. 1 and summarized in Table 1, PTEN protein expression was completely absent (score 0, - in Table 1) in 9/30 tumor cell lines; among 21 cell lines with PTEN protein expression, PTEN expression was weak (score 0.1–0.3, + in Table 1) in 10, moderate (score 0.3–0.6, ++ in Table 1) in 9, and strong (score 0.6–1, +++ in Table 1) in 2. Statistical analysis showed a moderate correlation between PTEN mRNA and protein expression levels (p = 0.038, Figure S1).

In order to define PTEN expression profile unequivocally, we considered cell lines with any degree of PTEN protein expression (score 0.1–1) in the absence of PTEN gene alterations as PTEN-competent, while cell lines carrying PTEN deletions or inactivating mutations or completely lacking PTEN protein expression (score 0) are referred to as PTEN-loss (see also Table S1).

### PTEN expression modulates sensitivity to MEK, but not to mTOR, inhibition

Tumor cell lines characterized for the mutational status of KRAS, BRAF, and PTEN (Table 1 and S1) were exposed to either the MEK inhibitor Trametinib or the mTOR inhibitor Everolimus (both at concentrations ranging from 0.1 to 1000 nM) for 72 h and half maximal inhibitory concentration (IC_50_) were derived, based on the assessment of cell viability (Table S1). Neither BRAF, KRAS or PTEN mutational status appeared to significantly influence response to Trametinib (p = 0.24, p = 0.10 and p = 0.15, respectively, Figure S2A) or Everolimus (p = 0.46, p = 0.48 and p = 0.79, respectively, Figure S2B). In order to ascribe a mechanistic role to PTEN expression in determining functional response to MEK or mTOR inhibition, we silenced PTEN expression by shRNA in the PTEN-competent melanoma cell line M14 (clone M14/shPTEN, Figure S2C insert) and overexpressed a functional PTEN in the PTEN-loss melanoma cell line WM115 (clone WM/PTEN, Figure S2D insert). PTEN silencing rendered M14 cells more resistant to Trametinib, as shown by both dose-response and growth curves (Figures S2C and S3A), with a slight shift in the IC_50_ at 72 h (from 0.3 nM in M14 to 1 nM in M14/shPTEN). In WM115 cells, stable transfection with a GFP-tagged PTEN construct, slowed down basal growth rate (doubling time ~40 h in WM115 and 51 h in WM/PTEN, respectively; Figure S3B) and rendered cells remarkably more sensitive to Trametinib-induced growth inhibition (IC_50_ 4000 nM versus 0.06 nM in WM115 and WM/PTEN, respectively; Figures S2D and S3B). Conversely, no striking differences were observed in response to Everolimus exposure in either model (Figures S2C,D and S3A,B).

From a molecular perspective, we analyzed the effects of Trametinib and Everolimus on the phosphorylation of key mediators of the PI3K and MAPK pathways (Figure S4). As expected, after 24 hours of treatment Trametinib efficiently blocked ERK phosphorylation, while Everolimus increased AKT T308 and S473 phosphorylation. However, no major qualitative differences were noted in terms of perturbation of signaling induced by MEK or mTOR blockade according to PTEN expression.

### Analysis of pharmacological interactions between MEK and mTOR inhibitors according to PTEN status

**Figure 2:**
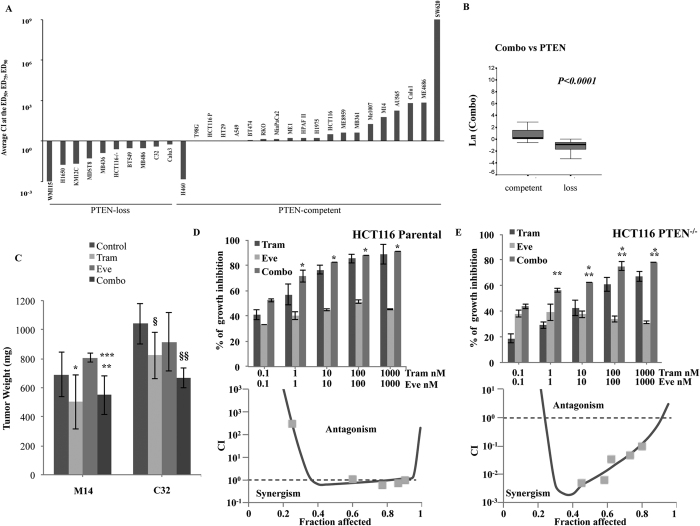
PTEN is a crucial determinant of synergism between MEK and mTOR inhibitors. (**A**) Growth inhibitory interactions between Trametinib and Everolimus were assessed in a panel of 30 tumor cell lines using a fixed-ratio (1:1) with a wide range of concentrations (0.1–1000 nM). Viability was then assessed after 72 h by Crystal violet assay and pharmacologic interactions were evaluated using the Calcusyn software. By this method, an average combination index (CI) at the ED_50_, ED_75_, and ED_90_ < 1 indicates synergism, = 1 indicates additivity, and >1 indicates antagonism. (**B**) Box plot shows the relationships between Trametinib/Everolimus pharmacologic interactions (CI) and PTEN status in a panel of 30 tumor cell lines. (**C**) Nude mice were injected i.m. with M14 (PTEN-competent) and C32 (PTEN loss) cells; when tumors became palpable, mice were treated with Trametinib 0.2 mg/Kg (Tram), Everolimus 2 mg/Kg (Eve) or their combination (Combo) for up 13 days. Differences in tumor weight after 7 (M14) or 10 (C32) days of treatment are shown. Tumor size was measured by caliper. Results from one representative experiment of at least two performed are shown and are expressed as average tumor weight (mg) ± SD for each treatment group. In M14-derived tumors: *p = 0.034 (by 2-tailed Student’s t test) for the comparison between control and Trametinib-treated, **p = 0.056 for the comparison between control and combination-treated, ***p = 0.001 for the comparison between Everolimus- and combination-treated; all other comparisons, including comparison between Trametinib- and combination-treated, were not significant. In C32-derived tumors: ^§^p = 0.006 for the comparison between control and Trametinib-treated ^§§^p < 0.01 for the comparison between control and combination-treated, Everolimus- and combination-treated, Trametinib- and combination-treated. (**D**,**E**) Isogenic HCT116 cell lines were treated with Trametinib and Everolimus, alone or in combination, using a fixed dose ratio (1:1). Cell viability was assessed by Crystal violet assay after 72 h. Results are expressed as percentage of growth inhibition relative to untreated control and represent the average ± SEM of three independent experiments (top). CI were plotted against the fraction affected (bottom). Asterisks indicate statistically significant differences (p < 0.05 by 2-tailed Student’s t test) for the comparison between *Everolimus- and combination-treated cells or **Trametinib- and combination-treated cells.

The effect of combined treatment with Trametinib and Everolimus, using a fixed dose-ratio (1:1) experimental design over a wide range of concentrations (0.1–1000 nM) of each agent, was assessed *in vitro* on the same panel of 30 human cell lines (Table S1). As shown in Fig. 2A, pharmacologic interactions between the two agents were almost invariably synergistic in cells with PTEN-loss, while combined MEK/mTOR inhibition resulted in a slightly additive/frankly antagonistic growth inhibitory response in PTEN-competent tumor cells, with the notable exception of the H460 lung cancer cell line, where combined treatment achieved strongly synergistic growth inhibition, despite the presence of an intact PTEN gene and protein. Overall, PTEN-loss (but not BRAF or KRAS mutations, p = 0.91 and p = 0.40, respectively, data not shown) was significantly associated with a synergistic interaction between Trametinib and Everolimus (p < 0.0001; Fig. 2B). Two representative examples of additive/antagonistic pharmacologic interactions between Trametinib and Everolimus in PTEN-competent (M14 and ME8959) and of synergistic growth inhibitory response in PTEN-loss (WM115 and C32) melanoma models are reported in Figure S5.

We also set out to confirm the pharmacologic interactions observed *in vitro* between Trametinib and Everolimus in xenograft models *in vivo* (Fig. 2C). In particular, established M14-derived (PTEN-competent) or C32-derived (PTEN-loss) xenografts were treated with Trametinib and Everolimus, alone or in combination, for up to 13 days and the effects on tumor growth were evaluated at the point of maximum tumor inhibition. In the M14-derived model Trametinib, used as single agent, significantly inhibited tumor weight (p = 0.03 versus control). In this context, Everolimus had no significant effect, as either single agent (p = 0.1 versus control) or in combination with Trametinib (p = 0.56 versus Trametinib alone). Conversely, in the C32-derived model the combination of Trametinib and Everolimus had a significantly greater tumor growth inhibitory effect, as compared to each treatment alone (p = 0.02 for the comparison between Trametinib and combination; p = 0.01 for the comparison between Everolimus and combination; Fig. 2C).

We next analyzed the effects of combined MEK/mTOR inhibition on the expression of key apoptosis regulators by immunoblotting (Figure S5C). In both M14 (PTEN-competent) and WM115 (PTEN-loss) we observed Bim induction, caspase 3/7 and PARP cleavage following treatment with Trametinib; however, the addition of Everolimus slightly increased caspase 3/7 and PARP cleavage only at the 24 h time point in M14, while substantially increased apoptosis induction in WM115 at both 24 and 48 hours, suggesting that synergistic effects of the combination in PTEN-loss contexts are due, at least in part, to apoptosis induction.

### PTEN status causally affects response to combined MEK/mTOR inhibition

The potentially causal role of PTEN in determining the functional outcome of combined MEK and mTOR inhibition was assessed in an isogenic colon cancer cell line model, differing only for PTEN status: Fig. 2D,E shows the growth inhibitory response (top panel) and pharmacologic interactions (expressed as combination index - CI - versus fraction affected, bottom panel) observed with Trametinib and Everolimus in the X-MAN™ isogenic HCT116 cell lines. The growth inhibitory effect of combination treatment was additive in HCT116 Parental (PTEN-competent, average CI at the ED_50_, ED_75_, and ED_90_: 1) and strongly synergistic in HCT116 PTEN^−/−^ (PTEN-loss, average CI at the ED_50_, ED_75_, and ED_90_: 0.25), respectively; similar results (additive/antagonistic interactions in HCT116 Parental and highly synergistic interactions in HCT116 PTEN−/−) were obtained using different Trametinib/Everolimus ratios (1:1, 100:1, or 1:100; Figure S6).

**Figure 3:**
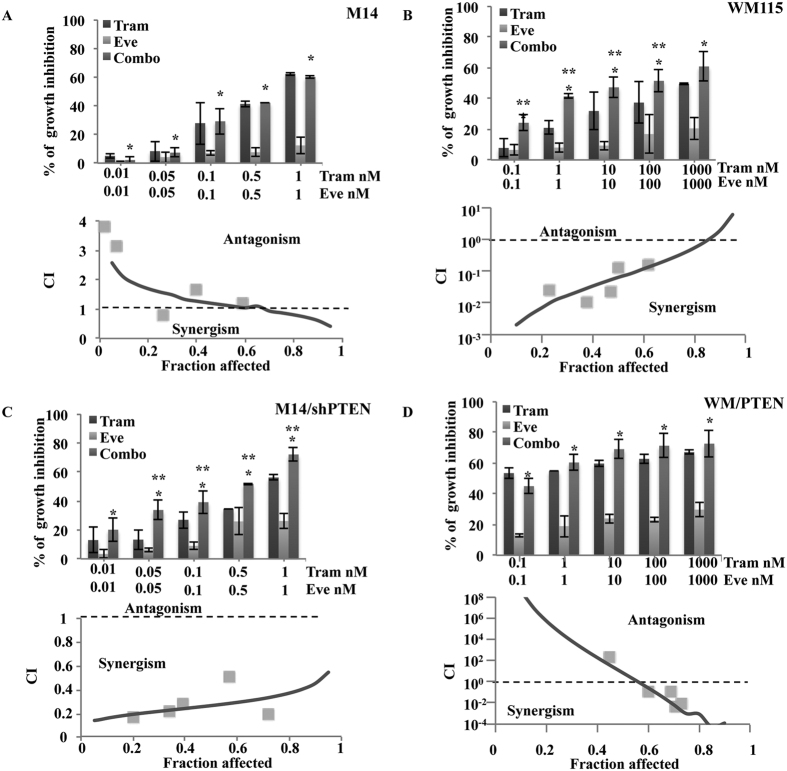
Genetic manipulation of PTEN expression modifies response to combined MEK/mTOR inhibition. (**A**–**C**) M14 clones stably transfected with either an empty plasmid vector (M14) or a plasmid encoding shPTEN (M14/shPTEN) were treated with Trametinib (Tram) and Everolimus (Eve), alone or in combination, using a fixed ratio (Combo 1:1). Cell viability was assessed by Crystal violet assay after 72 h. Results are expressed as percentage of growth inhibition relative to untreated control and represent the average ± SEM of three independent experiments (top). CI were calculated by conservative isobologram analysis for experimental data and plotted against the fraction affected (bottom). Asterisks indicate statistically significant differences (p < 0.05 by 2-tailed Student’s t test) for the comparison between *Everolimus- and combination-treated cells or **Trametinib- and combination-treated cells. (**B**–**D**) WM115 clones stably transfected with either an empty plasmid vector (WM115) or a plasmid encoding a GFP-tagged PTEN (WM/PTEN) were treated with Trametinib (Tram) and Everolimus (Eve), alone or in combination using a fixed ratio (Combo 1:1). Cell viability was assessed by Crystal violet assay after 72 h. Results are expressed as percentage of growth inhibition relative to untreated control and represent the average ± SEM of three independent experiments (top). CI were calculated by conservative isobologram analysis for experimental data and plotted against the fraction affected (bottom). Asterisks indicate statistically significant differences (p < 0.05 by 2-tailed Student’s t test) for the comparison between *Everolimus- and combination-treated cells or **Trametinib- and combination-treated cells.

In order to validate the role of PTEN status in determining the response to combined MEK and mTOR inhibition mechanistically, we performed combination experiments in M14/shPTEN and WM/PTEN (see also Figures S2 and S3). In the M14 model the combination of Trametinib and Everolimus did not afford increased growth inhibition, as compared to Trametinib alone (Fig. 3A, top panel). Conversely, in M14/shPTEN growth inhibition rates were significantly greater than those achieved with individual drugs (Fig. 3C, top panel). As a result, isobologram analysis indicated additive/antagonistic interactions in M14 cells and strong synergism between Trametinib and Everolimus in M14/shPTEN, with a CI of 1 and 0.3, respectively (Fig. 3A and C, bottom panels). On the other hand, combined Trametinib and Everolimus afforded significantly increased growth inhibition, as compared to each agent alone, resulting in a highly synergistic pharmacologic interaction in WM115 (Fig. 3B). In WM/PTEN, the introduction of a functional PTEN protein dramatically potentiated Trametinib’s growth inhibitory activity, which was only slightly increased by the addition of Everolimus (Fig. 3D, top panel). Although the experimental points fall in the highly synergistic part of the curve in both cellular models, the CI/fraction affected curve obtained in WM/PTEN actually mirrors that obtained in empty vector-transfected WM115 cells (Fig. 3B and D, bottom panels), suggesting, once again, that PTEN status dramatically influences response to combined MEK and mTOR inhibition.

### PTEN expression influences response to combined inhibition at different steps of the MAPK/PI3K cascades

**Figure 4:**
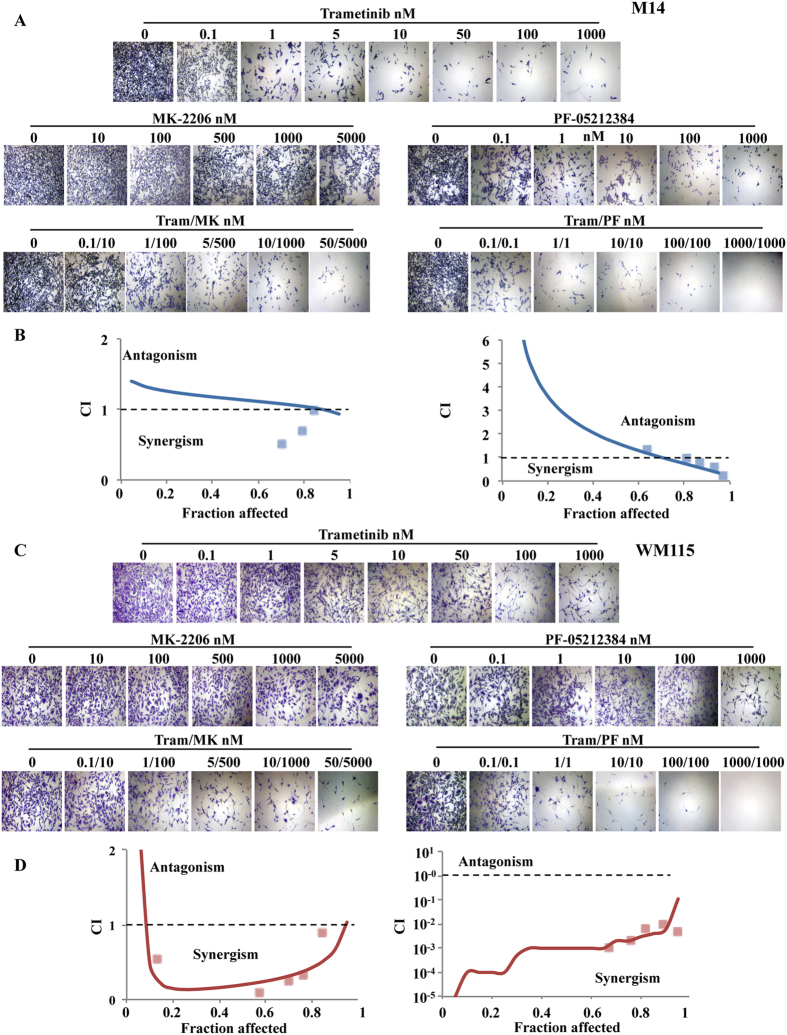
PTEN status affects response to MEK inhibition in combination with either AKT or double PI3K/mTOR inhibition. (**A**,**C**) M14 (PTEN-competent, **A**) and WM115 (PTEN-loss, **C**) cells were exposed to increasing concentrations of Trametinib (0.1–1000 nM) and either the allosteric AKT inhibitor MK-2206 (10–5000 nM) or the double PI3K/mTOR inhibitor PF-05212384 (0.1–1000 nM); combination experiments were performed using a fixed dose-ratio design using a 1:100 and 1:1 ratio, for Trametinib/MK-2206 and Trametinib/PF-05212384 combinations, respectively. Cells were exposed to treatments for 72 h and cell viability was assessed by Crystal violet assay (representative microscopic fields photographed are shown). Results of one experiment representing three independent experiments performed with superimposable results are shown. (**B**,**D**) CI were calculated by conservative isobologram analysis for experimental data and plotted against the fraction affected.

We next sought to ascertain whether the above-described findings on the influence of PTEN status on the functional outcome of combined pathway inhibition would specifically apply to the combination of MEK and mTORC1 inhibition. First, we substituted the selective BRAF inhibitor Dabrafenib for the MEK inhibitor Trametinib and tested it in combination with Everolimus in M14 and WM115 melanoma cells. Similar to the observations made with Trametinib, the combination of Dabrafenib and Everolimus exerted a synergistic growth-inhibitory effect only in PTEN-loss cells (WM115, CI: 0.7), while in the M14 model drug interaction was only additive (CI: 1) (Figure S7). We also tested a direct allosteric AKT inhibitor (MK-2206, dose-range 10–5000 nM) or a PI3K/mTOR double kinase inhibitor (PF-05212384, dose-range 0.1–1000 nM) in both M14 and WM115 cells, alone and in combination with Trametinib at a fixed dose-ratio (100:1 and 1:1, respectively). As shown in Fig. 4A,B, antagonistic/additive interactions were observed between MK-2206 and Trametinib (CI: 1) or PF-05212384 and Trametinib (CI: 1.1) in the PTEN-competent M14 cells, with slight synergism observed only with the combination PF-05212384/Trametinib at the highest concentrations. Conversely, the combination completely suppressed cell growth, resulting in a highly synergistic drug interaction (CI: 0.4 and 0.05 for MK-2206/Trametinib and PF-05212384/Trametinib combinations, respectively) in the PTEN-loss cell line WM115 (Fig. 4C,D, bottom). Similar results were obtained in ME8959 (PTEN-competent; CI: 1 and 13.7 for MK-2206/Trametinib and PF-05212384/Trametinib combinations, respectively) and C32 (PTEN-loss; CI: 0.5 for both MK-2206/Trametinib and PF-05212384/Trametinib combinations) melanoma cell lines (data not shown).

### Effects of combined MEK/mTOR inhibition in patient-derived CSC models

**Figure 5:**
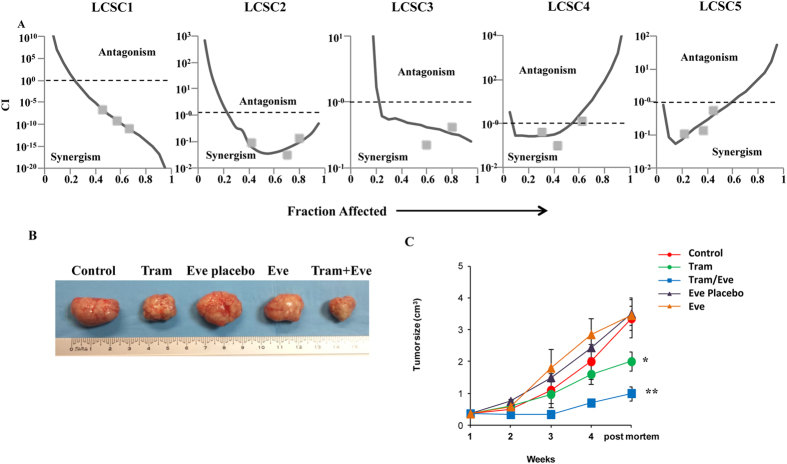
Effects of single and combined MEK and mTOR inhibition in LCSC. (**A**) Cells obtained from lung cancer spheres (LCSC) dissociation were plated in 96-well flat-bottom plates; Trametinib and Everolimus were added at their final concentration (1–1000 nM), as single agents or in a fixed dose-ratio combination (1:1). CI were calculated by conservative isobologram analysis for experimental data and plotted against the fraction affected. (**B**) Macroscopic image of subcutaneous tumor xenografts was obtained after injection of CR-CSCs. Mice were treated for 3 weeks with Vehicle or Placebo (Control or Eve placebo), Trametinib (Tram), Everolimus (Eve) alone or in combination (Tram/Eve). (**C**) Size of subcutaneous tumor xenografts was obtained in mice treated as in B. Data represents mean ± S.D. of three independent experiments. *p = 0.0009 for the comparison between control and Trametinib-treatment, **p for combined treatment with Trametinib and Everolimus as compared to either vehicle control (p = 0.0001) or single agent treatment (p = 0.003 for the comparison between Trametinib and combination, p = 0.0026 for the comparison between Everolimus and combination).

It has been reported that PTEN levels in cancer stem cells (CSC) are usually very low, as compared to more differentiated, “bulk” populations or established cell lines, particularly in lung and colorectal cancer-derived models (recently reviewed in ref. 19). Thus, we investigated the response of patient-derived melanoma initiating cells (MIC, n = 5) or lung CSC (LCSC, n = 5)^20^,^21^ to Trametinib and Everolimus, either alone or in combination, *in vitro* (Fig. 5A and S8A,B). All of the MIC analyzed expressed PTEN protein (Figure S8C) and were sensitive to Trametinib, but the addition of Everolimus had no appreciable effect (Figure S8A), so that pharmacological interactions were in the antagonistic range (CI: 2.4 to >10^9^, data not shown). Conversely, PTEN protein is expressed at very low levels in LCSC (ref. 22 and Figure S8C) and the combination of Trametinib and Everolimus resulted in a strikingly synergistic growth-inhibitory interaction *in vitro* in three out of five LCSC tested (LCSC1: CI = 7 × 10^−9^; LCSC2: CI = 0.11; LCSC3: CI = 0.34; Fig. 5A and S8B). We next tested the effects of Trametinib (0.3 mg/kg) and Everolimus (2 mg/kg), given as daily oral gavage either alone or in combination for up to 4 weeks, in a patient-derived colorectal CSC (CR-CSC) xenograft model *in vivo*; as shown in Fig. 5B,C, tumor growth was significantly inhibited by Trametinib exposure (p = 0.0009), while Everolimus-treated tumors did not significantly differ from vehicle control-exposed tumors in either their final size or their growth rate; however, combined treatment with Trametinib and Everolimus resulted in highly significant growth inhibition, as compared to either vehicle control (p = 0.0001) or single agent treatment (p = 0.003 for the comparison between Trametinib and combination; p = 0.0026 for the comparison between Everolimus and combination). *In vivo* treatment with Trametinib and Everolimus, alone and in combination was well tolerated, as no macroscopic signs of toxicity were observed and mice weight was conserved across treatment groups (data not shown).

### Proteomic Analysis

**Figure 6:**
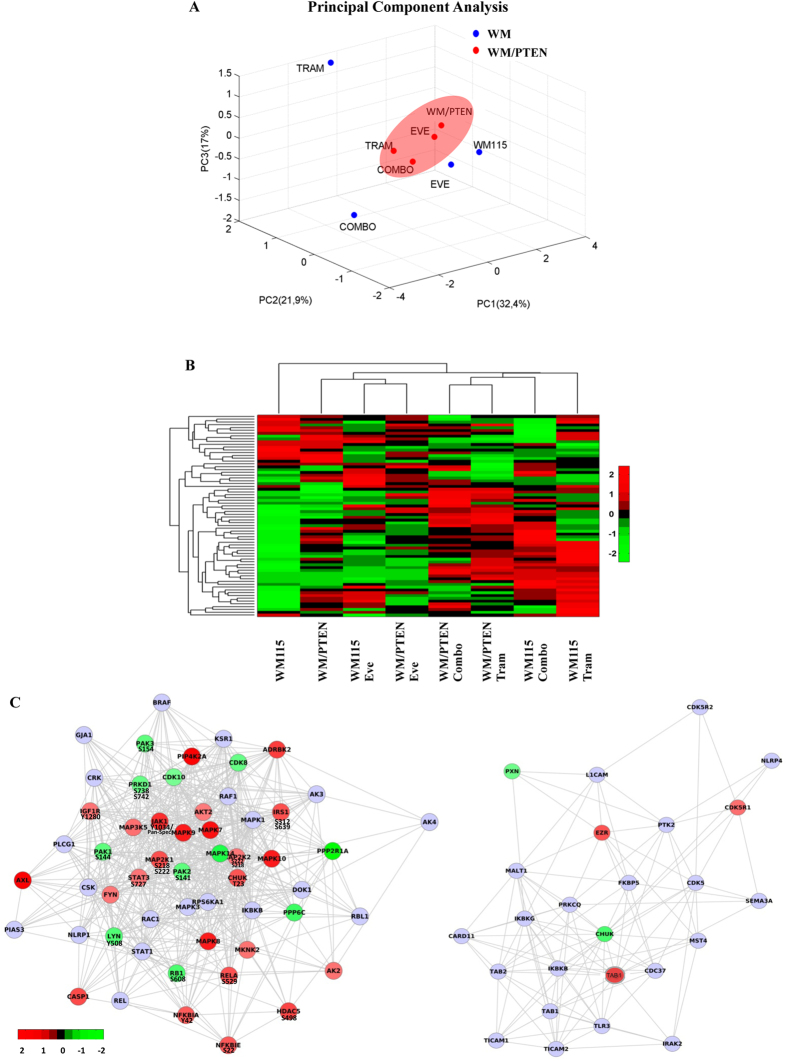
Proteomic analysis of WM115 (PTEN-loss) and WM/PTEN cells subjected to combined MEK and mTOR inhibition. WM115 cells stably transfected with either an empty plasmid vector (WM) or a plasmid encoding a GFP-tagged PTEN (WM/PTEN) were treated with Trametinib (100 nM), Everolimus (100 nM), or Trametinib + Everolimus (Combo) for 24 h. (**A**) Unsupervised Principal Component Analysis of all proteins deregulated by Trametinib and Everolimus, alone or in combination, in WM115 cells stably transfected with either an empty plasmid vector (WM) or a plasmid encoding a GFP-tagged PTEN (WM/PTEN). (**B**) Hierarchical Cluster of all deregulated proteins in at least one treatment using Trametinib (Tram), Everolimus (Eve), or the combination of the two drugs (Combo) in WM115and WM/PTEN cells. (**C**) Interaction network of the proteins found deregulated after combination treatment in WM115 and WM/PTEN. Network analysis was performed using Genemania; the resulting networks were then imported into Cytoscape in order to map fold-change values. Positive and negative fold changes are shown in red and green, respectively, on a scale from +2 to −2.

Protein expression profiles and changes in phosphorylation status at specific sites were assessed in WM115 and in WM/PTEN cells, after treatment with Trametinib, Everolimus or their combination, using antibody microarrays. A list of differentially expressed proteins identified as significantly modulated following at least one treatment is reported in Table S2. Principal Component Analysis (PCA) revealed more extensive treatment-induced modifications in total and phosphorylated protein expression in WM115 cells, as compared to WM/PTEN (Fig. 6A), particularly after treatment with single-agent Trametinib or the combination of Trametinib and Everolimus. Unsupervised hierarchical cluster analysis (heath map) of all proteins deregulated in at least one treatment is shown in Fig. 6B. Detailed heat maps of differentially expressed proteins are reported in Supplementary Figure S9: a limited number of total proteins significantly differed between untreated WM115 and WM/PTEN (Figure S9A), while no specific phosphorylation site appeared to be modulated by stable PTEN transfection. Single-agent Trametinib significantly affected the expression of 20 proteins (12 as total protein expression and 8 as phosphorylation status) and 13 proteins (10 as total protein expression as and 3 phosphorylation status) in WM115 and WM/PTEN, respectively, with no overlap in the affected proteins, except for CHUK and NFκBIα proteins which were affected by Trametinib treatment as phosphorylation status in WM115 and as total protein in WM/PTEN, respectively (Figure S9B and D). Single treatment with Everolimus significantly modulated the expression of 17 proteins (14 as total protein expression and 3 as phosphorylation status) and 3 proteins (as total protein expression) in WM115 and WM/PTEN cells, respectively, with no overlap (Figure S9C and E).

**Figure 7:**
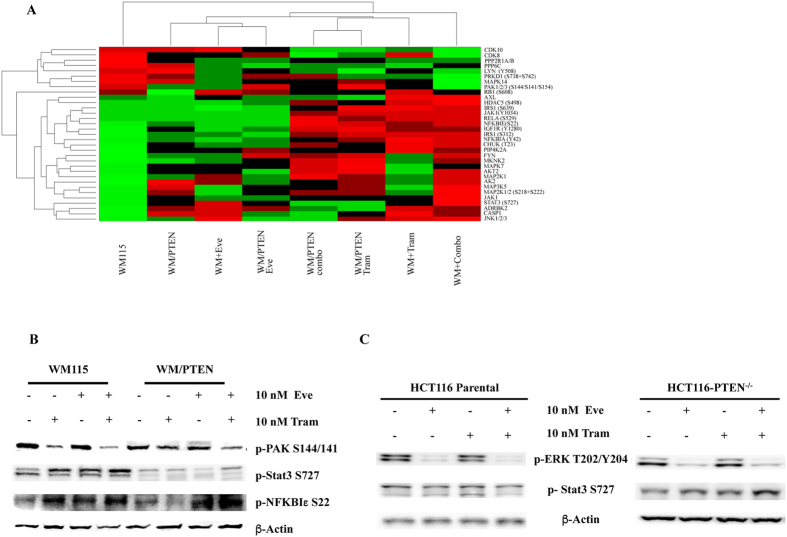
Hierarchical cluster of proteins deregulated after combination treatment in WM115 cell line. (**A**) Hierarchical cluster of proteins deregulated using combination treatment in WM115 and at the same time not deregulated with single-agent Trametinib or Everolimus treatments alone in both cell lines WM115 and WM/PTEN. (**B**) WM115 and WM/PTEN were treated with Trametinib (Tram), Everolimus (Eve), or the combination of the two drugs. The cells were lysed and analyzed by Western Blotting using antibodies specific for p-PAK1/2 (S144/S141), p-STAT3 (S727), p-NFκBIε (S22). Western blot with antibodies specific for β-actin are shown as protein loading and blotting control. (**C**) Isogenic HCT116 cell lines (HCT116 Parental and HCT116 PTEN^−/−^) were treated with Trametinib (Tram), Everolimus (Eve), or the combination of the two drugs and cell lysates were analyzed by Western Blotting using the indicated antibodies and β-actin was used as blotting control.

The effects of the combined treatment on protein expression profile in WM115 and WM/PTEN are shown in the network analysis in Fig. 6C. A considerable number of proteins were modulated in WM115, while relatively few changes were observed in WM/PTEN, suggesting that PTEN status can strongly influence the molecular response to combined treatment. Response to combined treatment also differed qualitatively, since the only protein modulated by the combination in both WM115 and WM/PTEN was CHUK, downregulated as total protein in both cell lines and phosphorylated on T23 in WM115 (Figure S9D). We next filtered and clustered data to highlight changes in proteomic profiles occurring exclusively under conditions in which combined treatment resulted in synergistic growth inhibition (i.e. changes occurring after combined treatment in WM115, but not in clone WM/PTEN, and not occurring after single-agent Trametinib/Everolimus treatment in either cell line, Fig. 7A). With this approach, the most significant changes were observed in components of the JAK1/STAT3 network (both total and phosphorylated proteins), phosphorylated PAK1/2/3, and phosphorylated NFκBIε. These results were validated by Western Blot assay: phosphorylated PAK1/2 (S144/141) was downregulated by Trametinib and combined Trametinib and Everolimus treatment more prominently in WM115 than in WM/PTEN; STAT3 phosphorylation (S727) was strongly induced by combination treatment in WM115 and to a much lesser extent in WM/PTEN; phosphorylated NFκBIε was upregulated by single and combined treatments in WM115 and only by Everolimus and combined treatment WM115/PTEN cells (Fig. 7B). Selective upregulation of STAT3 phosphorylation by combined MEK/mTOR inhibition in PTEN-loss contexts was also confirmed in isogenic HCT116 cell lines (HCT116 Parental and HCT116 PTEN^−/−^; Fig. 7C).

## Discussion

The general aim of our study was to assess the molecular determinants of therapeutic synergism between MAPK and PI3K pathway inhibitors. In a panel of human cancer cell lines of different histological origin, including isogenic colorectal cancer cell lines only differing for PTEN status, PTEN-loss effectively predicted synergistic growth inhibitory interactions between RAF/MEK and PI3K/AKT/mTOR inhibitors. Moreover, PTEN appeared to play a causal role in determining pharmacological interactions between pathway inhibitors, as genetic manipulation of PTEN expression in melanoma cell lines, dramatically altered the functional response to combined MEK/mTOR inhibition.

Combined inhibition of both the MAPK and PI3K pathways is being actively explored as an attractive therapeutic strategy in oncology. Although PTEN-loss has not been formally linked to synergistic pharmacologic interactions between MAPK and PI3K/mTOR inhibitors, careful analysis of published evidence suggests that such combination provides more striking tumor control in PTEN-loss, as compared with PTEN-competent, preclinical models. Indeed, Kinkade *et al*. have demonstrated that combinatorial blockade of MEK and mTOR signaling was highly synergistic, as compared to single-pathway inhibition, in terms of growth and proliferation inhibition of castration-resistant prostate cancer in the Nkx3.1, PTEN-mutant, mouse model^23^. Carracedo *et al*. simultaneously reported that combined MEK and mTOR inhibition was also effective in tumor xenografts generated using the PTEN-competent breast cancer cell line MCF7; however, the increase in apoptotic index and the decrease in the percentage of Ki67-positive cells in the combined treatment group was barely additive, as compared with PD0325901- and Everolimus- single-treatment groups, in such PTEN wild-type context^24^. In line with the concept that combined RAF/MEK/ERK and PI3K/AKT/mTOR inhibition is selectively synergistic in PTEN-loss contexts, Daphu *et al*. have demonstrated that combined therapy with the BRAF inhibitor Vemurafenib and the mTOR inhibitor Temsirolimus is highly synergistic, as compared to single-drug treatment, in cell lines derived from human melanoma brain metastases harboring both a BRAF mutation and PTEN-loss^25^. Moreover, data derived from a phase I clinical trial program indicate that combined MEK/mTOR targeting achieved relatively prolonged disease control in patients whose tumors harbored RAS/RAF alterations and simultaneous PTEN loss^14^,^26^.

Here we show that synergistic growth inhibitory effects are invariably observed with combined inhibition of the MAPK (using either BRAF or MEK inhibitors) and PI3K (using AKT, mTOR or double PI3K/mTOR inhibitors) pathways in cell lines lacking functional PTEN expression, suggesting that the growth inhibitory interactions occur at the pathway level, regardless of the specific point of the cascade being inhibited or of the MEK/mTOR inhibitor ratio used. Conversely, pharmacologic interactions between Trametinib and Everolimus are in the additive to antagonistic range in cells with an intact PTEN. The H460 lung cancer cell line constitutes a notable exception, as combined treatment resulted in striking synergistic growth inhibitory effects, despite the presence of an intact PTEN gene and protein. In this model, the presence of an LKB1/STK11 mutation may potentially explain these results: indeed, loss of LKB1 may lead to mTOR hyperactivation^27^,^28^, bypassing PTEN effects; moreover LKB1/STK11 and PTEN may interact with each other, resulting in LKB1 cytoplasmic retention and PTEN phosphorylation, although the biological significance of these modifications has not yet been defined^29^. Interestingly, LCSC1, in which combined MEK/mTOR is highly synergistic *in vitro*, also displays a LKB1/STK11 mutation. Analysis of the activation of apoptotic pathways shows that combined MEK/mTOR inhibition induces apoptosis more robustly and more persistently in PTEN-loss contexts, thereby partly explaining therapeutic synergism in this molecular context; however, we are currently investigating whether other growth inhibitory and/or cell death mechanisms (e.g. cell cycle inhibition, autophagy) also contribute to the observed tumor inhibitory synergism, particularly *in vivo*.

Genetic characterization of the melanoma, lung and colorectal cancer-derived CSC models tested revealed no obvious mutations in the PTEN gene. However, PTEN protein was consistently expressed in MIC, while it is very low, as compared to more differentiated, “bulk” populations or established cell lines, in LCSC and CR-CSC^19^,^22^,^30^,^31^,^32^; moreover, PTEN expression levels in CSC can be modulated upon differentiation and specific treatments (Eramo, 2016, unpublished results^10^,^19^,^30^,^31^,^32^). Overall, the relationship between PTEN gene status/protein expression and response to combined MEK/mTOR inhibition is less stringent in CSC models, possibly as a result of complex regulation mechanisms, encompassing transcriptional/post-transcriptional regulation, non-coding RNAs, post-translational modifications, protein-protein interactions, and sub-cellular localization; this exceptionally complex regulation makes it difficult to evaluate PTEN functional status in human tumors^19^,^33^,^34^,^35^. Although the role of PTEN expression/function in CSC needs to be studied in more detail, here we show that combined MEK/mTOR inhibition exerts synergistic anti-tumor activity *in vitro* and, most importantly, *in vivo*, in a proportion of lung and colorectal CSC-derived models. These results may be clinically relevant, as they raise the intriguing possibility that combined treatment could be key to selectively eliminate CSC populations, which are usually resistant to current therapeutic strategies^36^.

Although PTEN status profoundly affects functional response of cancer cells to single or combined MEK/mTOR blockade, PTEN silencing or enforced expression does not significantly alter pharmacologic response to Trametinib and Everolimus in terms of activation of obvious downstream targets along the respective signaling cascades (see Supplementary Figure S4 and data not shown). This suggests that important mediators of the synergistic interactions between Trametinib and Everolimus in a PTEN-loss context may actually lie outside the classical MAPK and PI3K pathways. Thus, we explored global changes in phosphoproteomic profiles by high-throughput proteomic analysis. The most extensive modifications in protein expression/phosphorylation profiles in response to single or combined MEK/mTOR inhibition occurred in cells lacking functional PTEN, whereas its enforced expression significantly blunted molecular response to treatments. Among proteins that are selectively modulated by combined, as compared with single-agent, treatment in the PTEN-loss WM115 cell line (in which combined treatment is functionally synergistic) we found PAK, NFκBIε, and kinases of the JAK/STAT (JAK1, STAT3, MAPK14) pathway of particular potential interest. PAK act as key signal transducers in several cancer signaling pathways, including RAS/RAF/MAPK and PI3K/AKT. Several studies have highlighted the role of PAK in the phosphorylation of both RAF-1 and MEK, facilitating signaling through the RAS/RAF/MAPK pathway^37^,^38^. On the other hand, PAK is a key mediator in the PI3K-AKT signaling axis through AKT phosphorylation on S473 and T308; furthermore, PAK could be directly activated by PI3K via RAC1/Cdc45^39^,^40^. Activation of the transcription factor NFκB appears to be a prominent mechanism by which PAK1 potentially regulates survival of cancer cells^40^,^41^; consistent with the hypothesis that combined MEK/mTOR inhibition in PTEN-loss contexts might synergistically inhibit tumor growth by selectively modulating a PAK/NFκB axis. Phosphorylation of the NFκB inhibitor NFκBIε^42^,^43^ was also modulated differentially by single or combined MEK/mTOR inhibition in PTEN-competent and PTEN-loss contexts. Recent studies have shown that in glioblastoma models STAT3 and PTEN activities, or lack thereof, may cooperate in modulating tumorigenesis and disease progression. Indeed, PTEN-mediated cooperative perturbation of AKT and STAT3 signals regulates proliferation and senescence in glioblastoma cells and the transcription factor STAT3 harbors a PTEN-regulated tumor suppressive function in mouse astrocytes^44^,^45^,^46^. Interestingly, NFκB and STAT3 extensively cross-regulate each other^47^ and synergistically control a common set of genes encoding for cytokines and chemokines^48^,^49^, such as IL-8, which may constitute a common target of the anti-tumor activity of combined MEK/mTOR inhibition in PTEN-loss contexts, particularly *in vivo*, through modulation of tumor/microenvironment interactions. Indeed, we have recently observed that IL-8 is overexpressed in a PTEN-loss context and downregulated in response to combined MAPK/PI3K inhibition in colorectal cancer models, possibly as a consequence of increased STAT3 phosphorylation (Ciuffreda, 2016, unpublished observation).

Overall, the results obtained clearly highlight a crucial role for PTEN in determining the functional outcome of combined MEK/mTOR inhibition. Such combination has shown substantial clinical toxicity in a recently completed phase I study^50^, where a recommended phase II dose and schedule of Trametinib in combination with Everolimus could not be identified. However, durable disease control was observed in approximately 30% of patients, suggesting that some patients may derive clinically significant benefit, even if treated with largely suboptimal single-agent doses. Our data suggest that PTEN status may potentially be developed as a biomarker of clinical situations in which combined inhibition of the MEK/ERK and PI3K/AKT/mTOR pathways could be highly synergistic and require reduced single-agent doses of each agent, thereby reducing toxicity. This is often the case, as combination drug therapies are usually more toxic than monotherapy alternatives, compromising quality of life, but also potentially providing survival advantages and reducing/delaying the development of drug resistance^51^,^52^. Indeed, the identification of the detailed molecular mechanisms and putative biomarkers of synergistic therapeutic interactions will be crucial to successfully develop combination strategies in the clinical setting, allowing for the selection/enrichment of patients at the highest chance of benefit^12^,^17^,^18^.

## Materials and Methods

### Cell lines and plasmid transfections

All cell lines were obtained from the American Type Culture Collection (ATCC), with the exception of WM115 cells, which were kindly donated by Dr. Meenhard Herlyn (Wistar Institute, Philadelphia, USA), and C32, KM12C and SW620, which were kindly donated by Dr. Federica Di Nicolantonio (University of Turin, Turin, Italy). X-MAN™ HCT116 Parental and HCT116 PTEN^−/−^ were generated by Horizon from homozygous knock-out of PTEN by deleting exon 5 which encodes the active site of the protein in the colorectal cancer cell line HCT116 (Horizon Discovery. www.horizondiscovery.com). Cell lines were routinely maintained in RPMI 1640 or DMEM medium supplemented with 10% fetal bovine serum (FBS), 2 mM L-glutamine, and antibiotics in a humidified atmosphere with 5% CO2 at 37 °C. Cell culture reagents were purchased from Invitrogen (Milan, Italy). Cells were transfected with either a GFP-PTEN expression construct^53^ or SureSilencing short hairpin RNA (shRNA) plasmids against PTEN (SABiosciences, Frederick, USA). Transfections were performed using the TransIT-LT1 transfection reagent (Mirus Bio LLC; Madison, USA) according to the manufacturer’s protocol.

More information about cell proliferation assay, drugs and cells and treatments are provided in Supplementary Experimental Procedures.

### Next-generation targeted sequencing

A custom panel to analyze all exons of PTEN gene was used. Details on target regions of panel are given in Supplementary Table S3 20 ng of DNA were used for each multiplex PCR amplification. The quality of the obtained libraries was evaluated by the Agilent 2100 Bioanalyzer on-chip electrophoresis (Agilent Technologies). Emulsion PCR was performed with the Ion OneTouch™ OT2 System (Life Technologies). Sequencing was run on the Ion Proton (PI, Life Technologies) loaded with Ion PI Chip v2. Data analysis, including alignment to the hg19 human reference genome and variant calling, was done using the Torrent Suite Software v.5.0 (Life Technologies). Filtered variants were annotated using a custom pipeline based on vcflib (https://github.com/ekg/vcflib), SnpSift1, the Variant Effect Predictor (VEP) software2 and NCBI RefSeq database. A baseline for CNV detection was performed using 10 normal male DNA extracted from fresh frozen tissues. Copy number variation analysis of gene was performed using adequate pipeline using IonReporter 5.0 version (Life Technologies).

### Melanoma, Lung and Colorectal cancer stem cells: isolation, culture and treatment

MIC and LCSC were derived from tumor samples obtained in accordance with procedures and protocols approved by the internal review board of the Sant’Andrea Hospital, University of Rome “La Sapienza”, as described elsewhere^20^,^21^,^22^,^54^. CR-CSC were obtained in accordance with procedures and protocols approved by the internal review board of Department of Surgical and Oncological Sciences (University of Palermo) as described elsewhere^31^,^32^. All patients signed an informed consent form. The use of such material was formally approved by the Ethical Committee/Institutional Review Board of the Regina Elena National Cancer Institute, National Institutes of Health prior and University of Palermo to the commencement of the study.

More information on CSC generation, culture, and validation, including antibodies and reagents is provided in Supplementary Methods.

### Xenografts

Female CD-1 nude (nu/nu) mice, 6 to 8 weeks old, were used (Charles River Laboratories, Calco, Italy). Mice were housed under pathogen-free conditions and all procedures involving animals and their care were in accordance with national and international law sand policies. Solid tumors were obtained by intramuscular injection of 5 or 7 × 10^6^ viable cells for M14 and C32, respectively. Each experimental group included 8 to 10 animals. Trametinib was formulated in 0.5% hydroxypropyl methylcellulose plus 0.2% Tween 80 and administered by oral gavage at the dosage of 0.2 mg/kg per day. Everolimus was administrated by gavage at the dosage of 2 mg/kg per day. Mice treated with an equal amount of vehicle were used as control groups. Treatment started when the tumor mass reached palpability (approximately 200 mg), and drugs were administered daily for 13 days; tumor size was measured every 2 to 3 days. We chose doses of Trametinib and Everolimus at the lower end of previously published evidence^55^,^56^ *in vivo* studies, in order to better assess therapeutic synergism and minimize toxicity in the combination group. Mice were killed when tumor volume reached more than 2000 mg, and tumors were excised and placed in 10% buffered formaldehyde. Tumor weight was calculated from caliper measurements according to the following formula: tumor weight (mg) = length (mm) × width (mm)2/2. In xenograft experiments carried out in CR-CSC-derived models Trametinib 0.3 mg/kg and Everolimus 2 mg/kg were used.

The three R’s principles were followed in planning and conducting all experiments involving live animals; the *in vivo* experimental plan was revised and formally approved by the Ethical Committee/Institutional Review Board of the Regina Elena National Cancer Institute and of the University of Palermo.

### Western blot analysis

In all cell lines tested, PTEN protein expression was assessed using Western blot assay. For western blotting, total cell lysates were prepared as described previously^57^. More information on this method including antibodies and reagents is provided in Supplementary Methods.

### Proteomic Analysis

For pan-specific and phosphosite-specific antibody microarray analysis, cells were lysed using a lysis buffer [1% NP 40, 20 mM HEPES (pH 7.4), and 2 mM EDTA] containing phosphatase inhibitors (100 mM NaF, 10 mM Na_4_P_2_O_7_, 1 mM NaVO_3_ and 1 mM molybdate), protease inhibitors (10 μM leupeptin, 10 μg/ml aprotinin, and 1 mM PMSF) and disulphide reducing agent (1 mM dithiothreitol). The final protein concentration in SDS-PAGE sample buffer was adjusted to 1 mg/ml. Samples were analyzed by Kinexus Bioinformatics Corporation (Vancouver, British Columbia, Canada). A complete list of unique target proteins and phospho-sites tracked in the microarray is available at http://www.kinexus.ca. More information about proteomic analysis can be found in Supplemental Experimental Procedures.

### Statistical Analysis

Differences between the groups were analyzed with a 2-tailed Student’s t test for paired samples. Associations were analyzed by Chi Square or Fisher’s Exact test, as appropriate. Correlations between treatment effects and genetic aberrations were analyzed according to Mann-Whitney (non parametric) tests. Significance was defined at the p < 0.05. The SPSS^®^ (21.0) statistical program was used for all analyses. Synergism, additivity, and antagonism were assessed by isobologram analysis with a fixed-ratio experimental design using the Chou-Talalay method^58^. Results were analyzed with the Calcusyn software (Biosoft, Cambridge, United Kingdom) and combination indexes (CI) were appropriately derived. By this method, an average CI at the ED_50_, ED_75_, and ED_90_ < 1 indicates synergism, = 1 indicates additivity, and >1 indicates antagonism, respectively.

All the experimental methods were performed in accordance with the institutional National and International guidelines and regulations.

## Additional Information

**How to cite this article**: Milella, M. *et al*. PTEN status is a crucial determinant of the functional outcome of combined MEK and mTOR inhibition in cancer. *Sci. Rep.* **7**, 43013; doi: 10.1038/srep43013 (2017).

**Publisher's note:** Springer Nature remains neutral with regard to jurisdictional claims in published maps and institutional affiliations.

## Supplementary Material

Supplementary Information

## Figures and Tables

**Table 1:** PTEN status in cancer cell lines.

Cell Line	MUT PTEN status	COSMIC ID	PTEN CNV	PTEN PTOTEIN EXPRESSION*	Relative PTEN mRNA abundance**
Melanoma					
WM115			LOH^§^	−	0.14
ME8959			LOH^§^	++	0.21
ME4686	Pro38Ser	COSM5142	LOH^§^	+	0.22
ME1007				++	0.62
M14				+	0.13
ME1				+	0.73
C32			LOH^§^	−	0.03
Breast					
BT474				++	0.06
BT549				−	0.15
AU565				+	0.02
MDA-MB468	253+1 G > T (splice site donor)	COSM13730		−	0.32
MDA-MB436			LOH^§^	−	0.51
MDA-MB361				++	0.88
Lung					
NCI-H1650			LOSS	−	0.04
NCI-H1975			GAIN (3)	++	0.65
Calu-1				+	0.63
Calu-3				+	0.1
NCI-H460				+++	0.26
A549				+	0.31
Colon					
KM12C	Gly129Ter; Lys267ArgfsTer9	COSM18663		−	0.20
SW620			GAIN (3)	++	0.41
HT29				++	0.40
MSDT8	Asp310Gly	COSM1968270		−	0.01
RKO				+	0.12
HCT116				++	0.35
HCT116 Parental				++	0.39
HCT116 PTEN^−/−^	frameshift_variant, stop_lost			−	0.23
Pancreas					
MiaPaCa				+	0.36
HPAF II				+	0.74
Glioblastoma Positive Control					
T98G	Leu42Arg	COSM5269		+++	1

*OD ratio of PTEN antibody/β-actin for each individual sample is compared with OD of positive control T98G. + score 0.1–0.3; ++ score 0.3–0.6; +++ score 0.6–1.

**Results represent PTEN mRNA abundance relative to positive control T98G.

Abbreviation used in the Table: LOH, Loss of heterozygosity.
