# Supplementary material for: PTEN status is a crucial determinant of the functional outcome of combined MEK and mTOR inhibition in cancer
Source: Sci Rep. 2017 Feb 21;7:43013. doi: 10.1038/srep43013 (PMC5318947; doi:10.1038/srep43013)
Supplement: Supplementary Information [file srep43013-s1.pdf]

Supplementary Informations

PTEN status is a crucial determinant of the functional outcome of combined MEK and mTOR inhibition in cancer

Michele Milella[1*§], Italia Falcone[1§], Fabiana Conciatori[1], Silvia Matteoni[1], Andrea Sacconi[2], Teresa De Luca[3], Chiara Bazzichetto[1], Vincenzo Corbo[7], Michele Simbolo[7], Isabella Sperduti[4], Antonina Benfante[8], Anais Del Curatolo[1], Ursula Cesta Incani[1], Federico Malusa[5], Adriana Eramo[6], Giovanni Sette[6], Aldo Scarpa[7], Marina Konopleva[9], Michael Andreeff[9], James Andrew McCubrey[10], Giovanni Blandino[2], Matilde Todaro[8], Giorgio Stassi[8], Ruggero De Maria[11], Francesco Cognetti[1], Donatella Del Bufalo[3], and Ludovica Ciuffreda[1*].

[1]Medical Oncology 1, Regina Elena National Cancer Institute, Rome, Italy; [2]Translational Oncogenomic Unit, Regina Elena National Cancer Institute, Rome, Italy; [3]Experimental Chemotherapy Laboratory, Regina Elena National Cancer Institute, Rome, Italy; [4]Biostatistics, Regina Elena National Cancer Institute, Rome, Italy; [5]Data Analysis Unit, Siena Biotech S.p.A. Siena, Italy; [6]Department of Hematology, Oncology and Molecular Medicine, Istituto Superiore di Sanità, Rome, Italy; [7]ARC-Net Research Centre and Department of Pathology, University of Verona, Verona, Italy; [8] DiBiMIS University of Palermo, Palermo, Italy;
[9]Section of Molecular Hematology and Therapy, Department of Leukemia, The University of Texas MD Anderson Cancer Center, Houston (TX), USA; [10]Department of Microbiology & Immunology, Brody School of Medicine, East Carolina University, Greenville (NC), USA; [11]Scientific Direction, Regina Elena National Cancer Institute, Rome, Italy.

Supplementary Figure Legends

Figure S1: Correlation plot between RNA and protein expression. (A) The basal mRNA expression level of PTEN (Control=1) was correlated with basal level of PTEN protein (Control=1) expressed in a panel of 30 cancer cell lines.

Figure S2: PTEN-loss is associated with relative resistance to the MEK inhibitor Trametinib. A-B. Box plots show the relationship between response (IC_{50}) of a panel of 30 tumor cell lines (see Table S1) exposed to either Trametinib (A) or Everolimus (B) at concentrations ranging from 0.1-1000 nM for 72 hours and BRAF, KRAS and PTEN aberrations (PTEN-competent and PTEN-*loss* indicates PTEN status according to the definition given in the text). **C**. M14 clones stably transfected with either an empty plasmid vector (M14) or a plasmid encoding shPTEN (M14/shPTEN) were analyzed by western blot using the indicated antibodies (insert). M14 and M14/shPTEN were exposed to increasing concentrations of Trametinib (top) or Everolimus (bottom) at concentrations ranging from 0.01 to 1 nM for 72 hours and then assessed for cell viability by trypan blue exclusion counting. **D**. WM115 cells were transfected with an empty plasmid vector (pEGFP-C2, WM) or a plasmid encoding a GFP-tagged PTEN (WM/PTEN). GFP-Tag identifies the transfected PTEN construct as detected by an anti-GFP antibody (insert). WM and WM/PTEN were exposed to increasing concentrations of Trametinib (top) or Everolimus (bottom) at concentrations ranging from 0.1 to 1000 nM for 72 hours and then assessed for cell viability by trypan blue exclusion counting. Results are expressed as percentage of viable cells relative to vehicle-treated control and represent the average±SD of three independent experiments.

Figure S3: PTEN context influences cell growth. A. The cells transfected with empty plasmid vector (M14) and the cells transfected with plasmid encoding a shPTEN (M14/shPTEN) were exposed to Trametinib (top) and Everolimus (bottom) (1-100 nM); their cell growth and viability were assessed by trypan blue exclusion test and counted using a Thoma chamber at indicated times.

B. WM115 cells transfected with an empty plasmid vector (pEGFP-C2, WM) or a plasmid encoding a GFP-tagged PTEN (WM/PTEN) were exposed to increasing concentrations of Trametinib (top) or Everolimus (bottom) at concentrations ranging from 1 to 100 nM fo for the indicated periods and then assessed by trypan blue exclusion test and counted using a Thoma chamber. Results are expressed as percentage of viable cells relative to vehicle-treated control and represent the average±SD of three independent experiments.

Figure S4: Molecular analysis in cells with or without PTEN protein expression. A-D. Parental cell lines (M14 and WM115) and their clones (M14/shPTEN and WM/PTEN) were treated with Trametinib and Everolimus for 24h to increasing doses (1-1000nM). The cells were lysed and analyzed by Western Blotting using antibodies specific for the protein above indicated. Western blot with β- actin specific antibody is shown as protein loading and blotting control.

Figure S5: PTEN status influences response to combination treatment A-B. M14 and ME8959 (PTEN-competent) cell lines and WM115 and C32 (PTEN-loss) cell lines were exposed to increasing concentrations Trametinib (0.1-1000nM) and Everolimus (0.1-1000nM) alone or in combination (fixed ratio 1:1) for 72h and cell growth were assessed by Crystal Violet assay. Combination Indexes (CI) were calculated by conservative isobologram analysis for experimental data and plotted against the fraction affected. **C.** M14 (left panel) and WM115 (right panel) are treated with Trametinib (Tram 100 nM), Everolimus (Eve 100nM), or the combination of the two drugs (Combo Ratio 1:1). The cells were lysed and analyzed by Western Blotting using specific antibodies indicated. Western blot with antibodies specific for β-actin are shown as protein loading and blotting control.

Figure S6: Analysis of drug combinations according to PTEN status. The X-MAN™ isogenic cell lines HCT116 Parental (PTEN-competent, **A**) and HCT116 PTEN$^{-/-}$ (PTEN-loss, **B**) were

exposed to increasing concentrations of Trametinib (T) and Everolimus (E), alone or in combination at 1:1 (left panels), 100:1 (middle panels), and 1:100 T/E (right panels) ratios for 72h; the range of concentrations used for each agent were different for the 100:1 T/E ratio (T: 1-1000 nM; E: 0.01-10 nM) or 1:100 T/E ratio (T: 0.01-10 nM; E: 1-1000 nM); the full range of concentrations was tested at the 1:1 T/E ratio (0.01-1000 nM for both T and E). Cell growth was assessed by Crystal Violet assay and Combination Indexes (CI) were calculated by conservative isobologram analysis for experimental data and plotted against the fraction affected. CI/fraction affected plots were derived after averaging the results in terms of cell growth inhibition of three independent experiments.

Figure S7. Isobologram of combination index as a function of affected fraction. **A-B.** After 72h of treatment with Dabrafenib (0.01μM-10μM) and Everolimus (1nM-1000nM), alone or in combination (fixed ratio 100:1), cell growth and viability of M14 and WM115 were determined by Crystal Violet assay. Combination Indexes (CI) were calculated by conservative isobologram analysis for experimental data and plotted against the fraction affected.

Figure S8. **Effects of single and combined MEK and mTOR inhibition in CSC. A.** Dose-dependence curve of viability inhibition effect calculated after 72h of treatment of Melanoma Cancer Stem Cells (MCI) clones with scalar doses of Trametinib, Everolimus and their combination (range from 1-1000 nM). Results of one experiment representative of three independent experiments performed with superimposable results are shown. **B.** Cells obtained from lung cancer spheres (LCSC) dissociation were plated in 96-well flat-bottom plates; Trametinib and Everolimus were added at their final concentration (1-1000 nM), as single agents or in a fixed dose-ratio combination (1:1). Cell viability was evaluated after 3 days of treatment by both luminescent cell viability assay (CellTiter-Glo, Promega, Madison, WI, USA) and cell count by trypan blue exclusion. Results are expressed as percentage of viable cells relative to vehicle-treated control and

represent the average±SD of three independent experiments. **C**. Lysates obtained from MIC and LCSC clones were then subjected to Western Blotting with PTEN specific antibody. Protein levels were normalized using β-actin. T98G and U251 glioblastoma cell line were used, respectively, as PTEN positive and negative control cell lines. Asterisks indicate clone not analyzed in current submission. Results of one experiment representative of three independent experiments performed with superimposable results are shown.

Figure S9: Hierarchical Cluster Analysis. A. Heat map of differentially expressed proteins between untreated WM115 Empty Vector (EV) and PTEN Clone WWT4 cell lines. **B.** Proteins deregulated in WM115 cells, but not in WWT4, after treatment with Trametimib. **C.** Proteins deregulated in WM115 cells, but not in WWT4, after treatment with Everolimus. **D.** Proteins deregulated in WWT4 cells, but not in WM115, after treatment with Trametimib. **E.** Proteins deregulated in WM115 cells, but not in WWT4, after treatment with the combination and not deregulated by Trametinib or Everolimus alone.

Figure S10 Original western blots of Figure 7B and Figure S5C.

Supplementary Materials

Drugs treatment, cell proliferation assay and gene status

Trametinib (GSK1120212) and Dabrafenib (GSK2118436) were kindly provided by GlaxoSmithKline (Brentford, Middlesex, UK). Trametinib was dissolved in DMSO as a 1 mM stock solution and stored at -20°C. Dabrafenib was dissolved in DMSO as a 10 mM stock solution and stored at -20°C. Everolimus (RAD001) was obtained from Novartis Pharma (Basel, Switzerland) and was dissolved in 100% ethanol as a 10 mM stock solution and stored at -20°C. The final concentration of both drugs was obtained by dilution with culture medium. PF-05212384 was kindly provided by Pfizer Inc. (New York, NY, USA). MK-2206 was kindly provided by Merck and Co. (Kenilworth, NJ, USA). PF-05212384 and MK-2206 were dissolved in DMSO as a 1 mM stock solution and stored at -20°C.

Cell proliferation was evaluated by direct cell counting or Crystal Violet assay.

For cell counting, exponentially growing cells were exposed to different treatments for various periods of time (24 to 144 hours), washed, assayed for cell viability (by trypan blue exclusion test) and counted using a Thoma chamber.

For Crystal Violet assay, tumor cells were dispensed into 24-wells (Corning, Inc.) at a concentration of 2×10^4 cells/well. The following day drugs were added at indicated concentrations. After the incubation time the cells were washed three times with PBS, fixed with 4% (vol/vol) Formaldehyde, and stained with 0.1% crystal violet (CV) for 40 min at room temperature (RT). Excess stain was removed with water and the plates were dried. CV stain was extracted with 95% (vol/vol) acetic acid and the absorbance was measured at 570 nm.

The IC50 value was calculated according to the Chou-Talalay method using the Calcusyn software (Biosoft, Cambridge, United Kingdom***).***

In following cell lines: C32, NCI-H1650, NCI-H1975, A549, Calu-1, Calu-3, NCI-H460, MIA PaCa-2 and HPAF II, BRAF, NRAS, KRAS genes were performed by PCR-Sanger sequencing.

Melanoma, Lung and Colorectal cancer stem cells: isolation, culture and treatment

Lung cancer stem cells (LCSC) were isolated as described previously (Eramo, 2008) from tumor samples through selective culture in serum-free medium containing EGF 20 ng/ml and basic FGF 10 ng/ml (PeproTech, London, UK). LCSC from surgically resected tumor samples through selective culture in serum-free medium containing EGF 20 ng/ml and basic FGF 10 ng/ml (PeproTech, London, UK). Non-treated polystyrene flasks (Nunc, Thermo Fischer Scientific, Rochester, NY, USA) were used to reduce cell adherence and support growth of LCSC as undifferentiated tumor spheres. Cultures were expanded by mechanical partial dissociation of spheres, followed by re-plating of cells and residual small aggregates in complete fresh medium.

In vitro generation of melanoma initiating cells (MIC) was obtained as previously reported for glioblastoma and lung cancer (Eramo, 2008; Sette, 2013). Briefly, tumor samples were dissociated by enzymatic digestion (1.5mg/ml Collagenase II, Gibco-Invitrogen, Carlsbad, CA and 20 µg DNAse I, Roche, Mannheim, Germany) for 2 hours at 37°C and cell suspension washed and cultured in serum free stem cell-suitable medium containing nutrients and supplemented with 20 ng/ml EGF and 10 ng/ml bFGF (Eramo, 2006). Under these conditions cell growth of undifferentiated tumor cells occurred as spheroids and yielded highly tumorigenic MIC clones. These clones were validated for CSC properties using *in vitro* and *in vivo* assays (Sette, 2013).

Three thousand cells obtained from lung and melanoma cancer spheres dissociation were plated in 96-well flat-bottom plates. Trametinib and Everolimus were added at their final concentration, as single agents or in combination. Cell viability was evaluated after 3 days of treatment by both luminescent cell viability assay (CellTiter-Glo, Promega, Madison, WI, USA) and cell count by trypan blue exclusion.

Western blot analysis

The proteins were fractionated by SDS-polyacrylamide gel electrophoresis and transferred to nitrocellulose membrane (Amersham, Arlington Heights, USA). Membranes were probed with primary antibodies and the signal was detected using peroxidase-conjugated anti-mouse or anti-rabbit secondary antibodies (Jackson Immunoresearch Labs, Inc., Baltimore, USA). The enhanced chemi-luminescence (ECL) system (Amersham) was used for detection. The following primary Abs were used: phosphorylated (Ser473 and Thr308) and total AKT, phosphorylated (Thr202/Tyr204) and total ERK1/2, PTEN, phosphorylated (Ser371/Thr389) p70S6k, phosphorylated (Thr37/46) and total 4EBP1, phosphorylated (Ser727) STAT3, phosphorylated (Ser144/141) PAK1/2, phosphorylated (Ser22) NFκBIε, GFP, cleaved and total PARP, cleaved and total Caspase 3 and Caspase 7 and Bim (from Cell Signaling Technology Inc. Beverly, USA).

Image detection was performed with both UVITEC Alliance 4.7 system (Cambridge, UK) (UVITEC, Figure1, Figure S4, Figure 7) or with Amersham Hyperfilm ECL (Amersham, Figure S5C). To control the amount of proteins transferred to nitrocellulose membrane β-actin was used and detected by anti β-actin mAb (clone AC-15, Sigma, St. Louis, USA).

RNA analysis

Total RNA was prepared from cells using RNA extraction kit (Geneaid) as per the manufacturer's instructions. Of total RNA, 1 μg was converted into single-strand cDNA using Superscript II (Invitrogen) as per the manufacturer's instructions. Quantitative real-time PCR (qRT-PCR) was performed with Fast SYBR®Green quantitative PCR kit (Applied) for RPL19 (Forward primer sequence: 5'-CGGAAGGGCAGGCACAT-3' and Reverse primer sequence 5'-GGCGCAAAATCCTCATTCTC-3') and PTEN (Forward primer sequence: 5' AATAAAGACAAAGCCAACCGATACTT 3' and Reverse primer sequence 5' CGGCTCCTCTACTGTTTTTGTGA 3'). Expression of PTEN mRNA was then normalized with RPL19 and compared with mRNA positive control of T98G.

Proteomic Analysis

Kinexus' KinexTM 850-Antibody Microarray Kit is an array functionalized with more than 850 antibodies with the potential to detect signaling proteins that are altered in their expression, phosphorylation, or protein-protein interactions. The 850-Antibody Microarray includes approximately 517 pan- specific antibodies (for protein expression) and 337 phospho-site-specific antibodies (for phosphorylation).

Each microarray consists of 2 identical fields, allowing two samples to be analyzed side by side at a time. 50 μg of lysate protein from each sample are covalently labeled with a proprietary fluorescent dye, incubated on the array and images acquired using a laser scanner (Waltham, MA). Each sample was analyzed in duplicate. Images are captured with a Perkin-Elmer ScanArray Reader laser array scanner (Waltham, MA). Signal quantification and background correction were performed with ImaGene 8.0 from BioDiscovery (El Segundo, CA).

Bioinformatic signal analysis and feature selection were performed by Matlab (The MathWorks Inc.). Z score transformation was used to express the background corrected spot intensity values as unit of a standard deviation from the normalized mean of zero. Features were selected basing on Z ratios calculated by taking the difference between the averages of the observed protein Z scores and dividing by the standard deviation of all the differences for that particular comparison. A Z ratio higher than 1.3 was inferred as significant. Unsupervised Principal Component Analysis and Hierarchical Clustering were used to investigate clusters of samples. Interaction network analysis and functional association of deregulated proteins were performed using GeneMania web tool (www.genemania.org/) and Z-ratio values were integrated to the network in Cytoscape (www.cytoscape.org/) using color gradients into an arbitrary scale from -3 to +3 (red for the negative z-ratio values and green for the positive values). Pathway was customized using Path Designer tool of Ingenuity Pathway Analysis software (IPA; Ingenuity® Systems Ingenuity Pathway Analysis, www.ingenuity.com).

Changes in proteins and phosphorylation events that are short-listed and recommended for follow-up can be found in the second sheet, in the KinexTM Report named “Shortlist”.

The proteins are ranked by Z-Ratios. A negative number infers a decrease in expression or phosphorylation from the control sample, whereas a positive number infers an increase from the control. The parameters used for the short-listing of the proteins are Z-ratios, % Error Range, Flag and the median Globally Normalized Signal Intensity value.

Validation of key antibody microarray results obtained by an alternative approach such as Western blotting is therefore essential before significant resources are expended in following up on leads.

Table S1. Summary of the genetic status of the cell lines analyzed and response to treatments

Cell lines	Histology	*BRAF*	*NRAS/ KRAS*	PTEN status	Tram IC_{50} (nM)	Eve IC_{50} (nM)	CI
**WM115**	Melanoma	V600D	wt/wt	Loss	13	3×10^7	0.0005
**ME8959**	Melanoma	wt	Q61R/wt	Competent	0.6	7.7×10^6	4.21
**ME4686**	Melanoma	V600E	wt/wt	Competent	4728	9×10^{10}	742
**ME1007**	Melanoma	wt	wt/wt	Competent	6	37918	18.5
**M14**	Melanoma	V600E	wt/wt	Competent	3	6069	59
**ME1**	Melanoma	V600E	wt/wt	Competent	0.013	1518	1.6796
**C32**	Melanoma	V600E	wt/wt	Loss	177	5×10^8	0.3772
**BT549**	Breast	wt	wt/wt	Loss	21741	30	0.2814
**BT474**	Breast	wt	wt/wt	Competent	700	15	1.2146
**MDA-MB361**	Breast	wt	wt/wt	Competent	8337	2437	4.380
**MDA-MB468**	Breast	wt	wt/wt	Loss	57	3238	0.3008
**MDA-MB436**	Breast	wt	n.a./n.a.	Loss	443	371	0.1276
**AU565**	Breast	wt	wt/wt	Competent	1633	272	187
**NCI-H1650**	Lung	wt	wt/wt	Loss	50	33299	0.0177
**NCI-H1975**	Lung	wt	wt/wt	Competent	1.5	10	1.719
**A549**	Lung	wt	wt/G12S	Competent	1	80	1.0969
**Calu-1**	Lung	wt	wt/G12C	Competent	60	1×10^5	634
**Calu-3**	Lung	wt	wt/wt	Competent	144	1066	0.5322
**NCI-H460**	Lung	wt	wt/Q61H	Competent	354	2	0.0014
**KM12C**	Colon	wt	wt/wt	Loss	40	2×10^6	0.0200
**SW620**	Colon	wt	wt/G12V	Competent	10	4×10^5	8×10^9
**HCT 116**	Colon	wt	wt/G13D	Competent	1.7	4×10^5	3.1208
**HCT116 parental**	Colon	wt	wt/G13D	Competent	0.21	370	1
**HCT116 PTEN$^{-/-}$**	Colon	wt	wt/G13D	Loss	47	3×10^8	0.25
**HT-29**	Colon	V600E	wt/wt	Competent	0.008	130	1.101
**MDST8**	Colon	V600K	wt/wt	Loss	15	0.2	0.0536
**RKO**	Colon	V600E	wt/wt	Competent	1	470	1.3429
**MIA PaCa-2**	Pancreas	wt	wt/G12C	Competent	5.4	41	1.4371
**HPAF II**	Pancreas	wt	wt/G12D	Competent	3.7	5×10^8	1.7150
**T98G**	Glioblastoma	n.a.	n.a	Competent	4574	1.7	1

§BRAF, NRAS, KRAS, gene status was assessed by Sanger sequencing

Abbreviations used in the Table: Tram: Trametinib; Eve: Everolimus; CI: combination index; IC_{50}: concentration inhibiting cell growth by 50%; Ref: references; n.a.: not assessed

TableS2. List of proteins significantly modulated by at least one treatment

Target protein name	Phospho Site (Human)
AK2	Pan-specific
RIPK4	Pan-specific
MAP3K5	Pan-specific
AXL	Pan-specific
BAX	Pan-specific
BLK	Pan-specific
CA9	Pan-specific
CASP1	Pan-specific
CTNNB1	Pan-specific
CDK10	Pan-specific
CDK5	Pan-specific
CDK8	Pan-specific
ERBB2	Pan-specific
MAPK7	Pan-specific
EZR	T567
FYN	Pan-specific
GAB1	Y627
ADRBK2	Pan-specific
HDAC5	S498
HSP90AB1	S254
IGF1R	Y1280
IGF1R	Y1165/Y1166
NFKBIA	Pan-specific
NFKBIA	Y42
NFKBIE	S22
CHUK	Pan-specific
CHUK	T23
IRS1	S312
IRS1	S639
JAK1	Pan-specific
JAK1	Y1034
MAPK9 1/2/3	Pan-specific
JUN	Y170
LYN	Y508
MAP2K1	Pan-specific
MAP2K1/2	S218+S222
MAP3K4	Pan-specific
MKNK2	Pan-specific
STK4	Pan-specific
PPP1R12A	T696
NEK2	Pan-specific
RELA	S529
CDK5R1	Pan-specific
MAPK14	Pan-specific
TP53	S392
TP53	S33
PAK1	Pan-specific
PAK1/2/3	S144/S141/S154
PXN	Pan-specific
PDPK1	Pan-specific
PIK3R4	Pan-specific
PI4KB	Pan-specific
PIP4K2A	Pan-specific
PRKAR2A	Pan-specific
AKT2	Pan-specific
PRKD1	S738+S742
PPP1CB	Pan-specific
PPP2R1A/B	Pan-specific
PPP6C	Pan-specific
PTPN12	Pan-specific
PTK2	Pan-specific
RB1	S608
RPS6KA1	Pan-specific
RPS6KB1	Pan-specific
STAT1	Pan-specific
STAT2	Pan-specific
STAT3	S727
TAK1	Pan-specific
MAPT	S519
TNFSF10	Pan-specific
YES1	Pan-specific
ZAP70	Pan-specific

Supplementary Table 3 - Panel Design			
**Chr**	**Start**	**End**	**Gene**
**chr10**	89624204	89624323	PTEN
**chr10**	89653752	89653818	PTEN
**chr10**	89653817	89653930	PTEN
**chr10**	89685245	89685357	PTEN
**chr10**	89690820	89690917	PTEN
**chr10**	89692714	89692819	PTEN
**chr10**	89692820	89692920	PTEN
**chr10**	89692921	89693032	PTEN
**chr10**	89711803	89711928	PTEN
**chr10**	89711917	89712027	PTEN
**chr10**	89717580	89717695	PTEN
**chr10**	89717695	89717792	PTEN
**chr10**	89720564	89720686	PTEN
**chr10**	89720693	89720769	PTEN
**chr10**	89720770	89720842	PTEN
**chr10**	89724949	89725061	PTEN
**chr10**	89725059	89725147	PTEN
**chr10**	89725120	89725240	PTEN

Figure S1

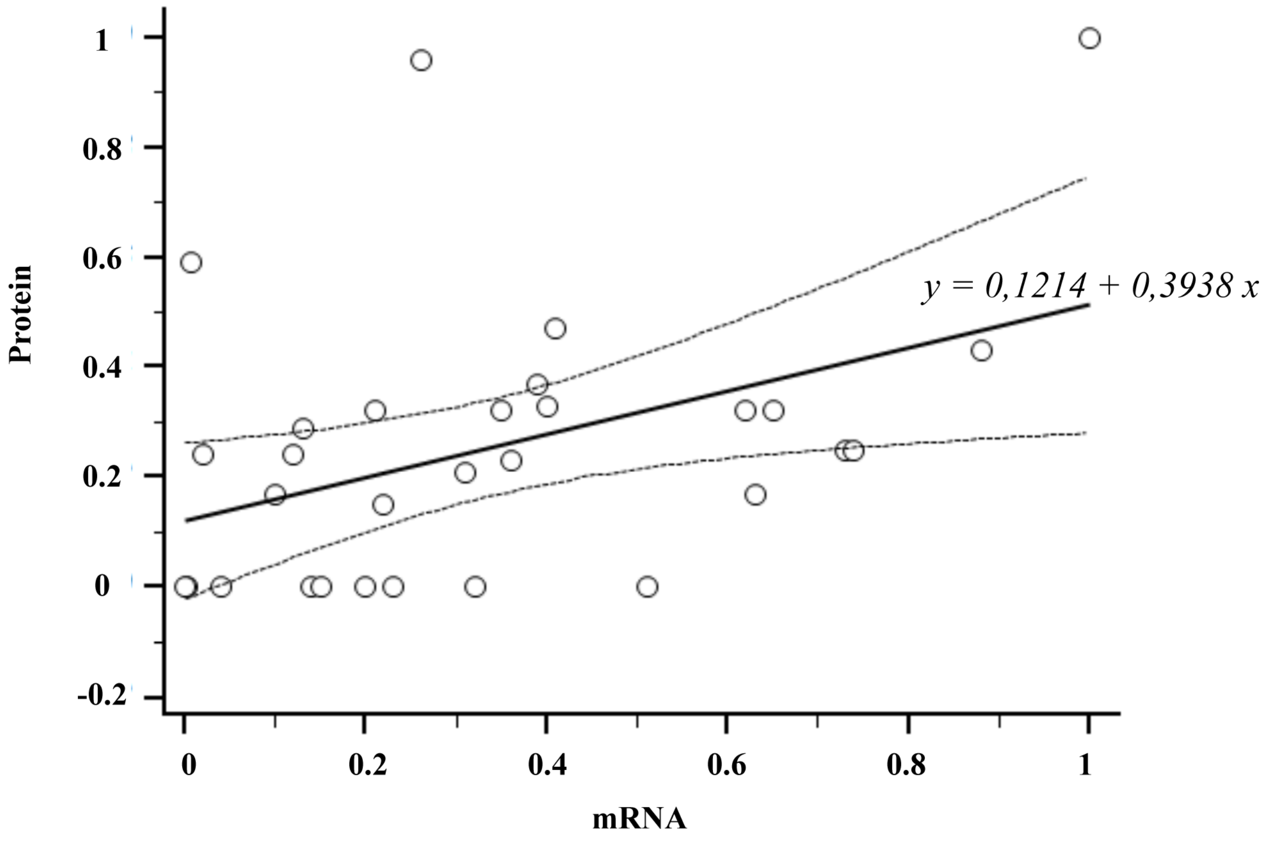

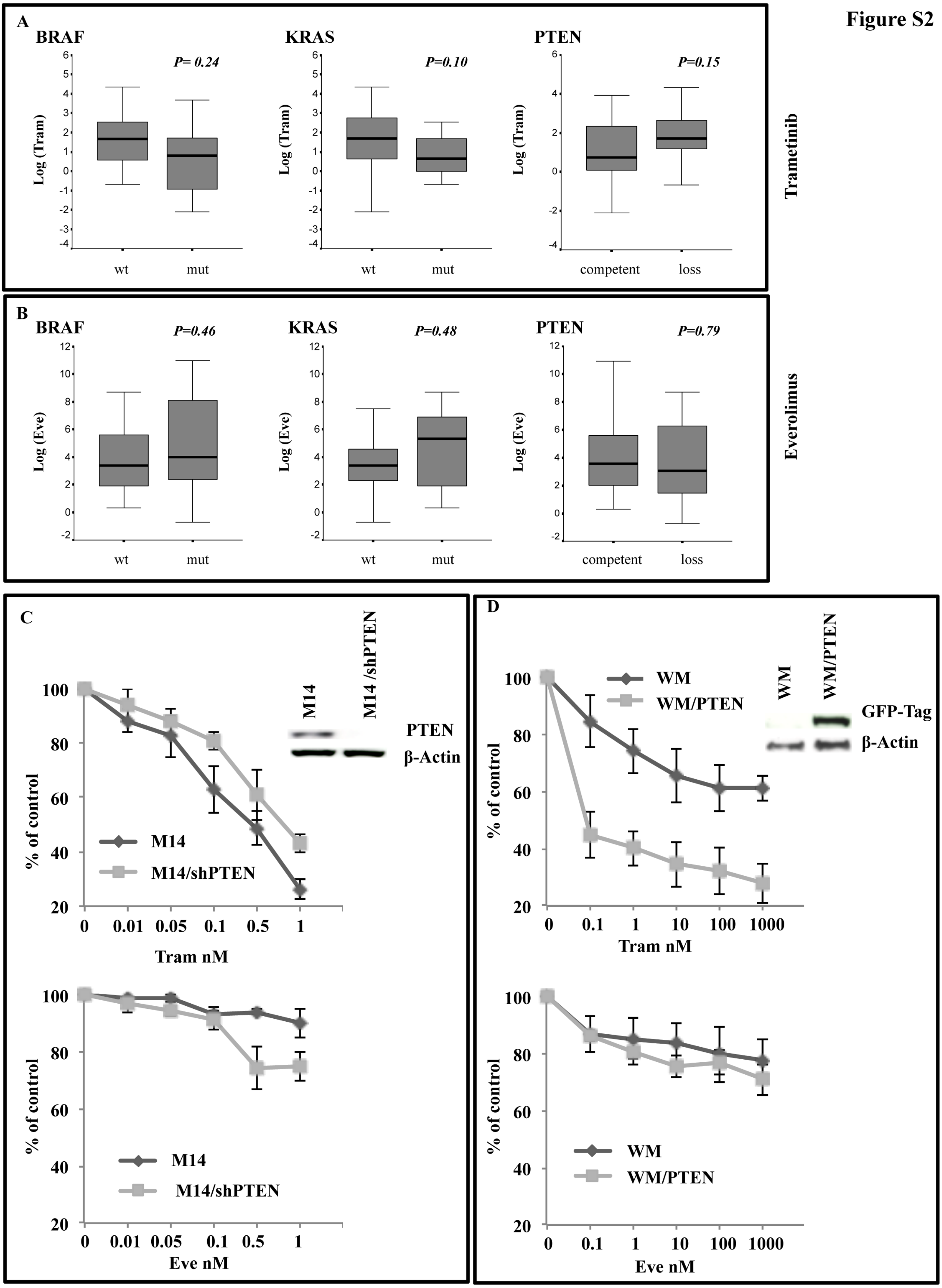
Figure S2
A
BRAF
P= 0.24
KRAS
P=0.10
PTEN
P=0.15
Log (Tram)
wt
mut
competent
loss
Trametinib
B
BRAF
P=0.46
KRAS
P=0.48
PTEN
P=0.79
Log (Eve)
wt
mut
competent
loss
Everolimus
C
M14
M14 /shPTEN
PTEN
β-Actin
% of control
M14
M14/shPTEN
Tram nM
Eve nM
D
WM
WM/PTEN
GFP-Tag
β-Actin
% of control
Tram nM
Eve nM

Figure S3

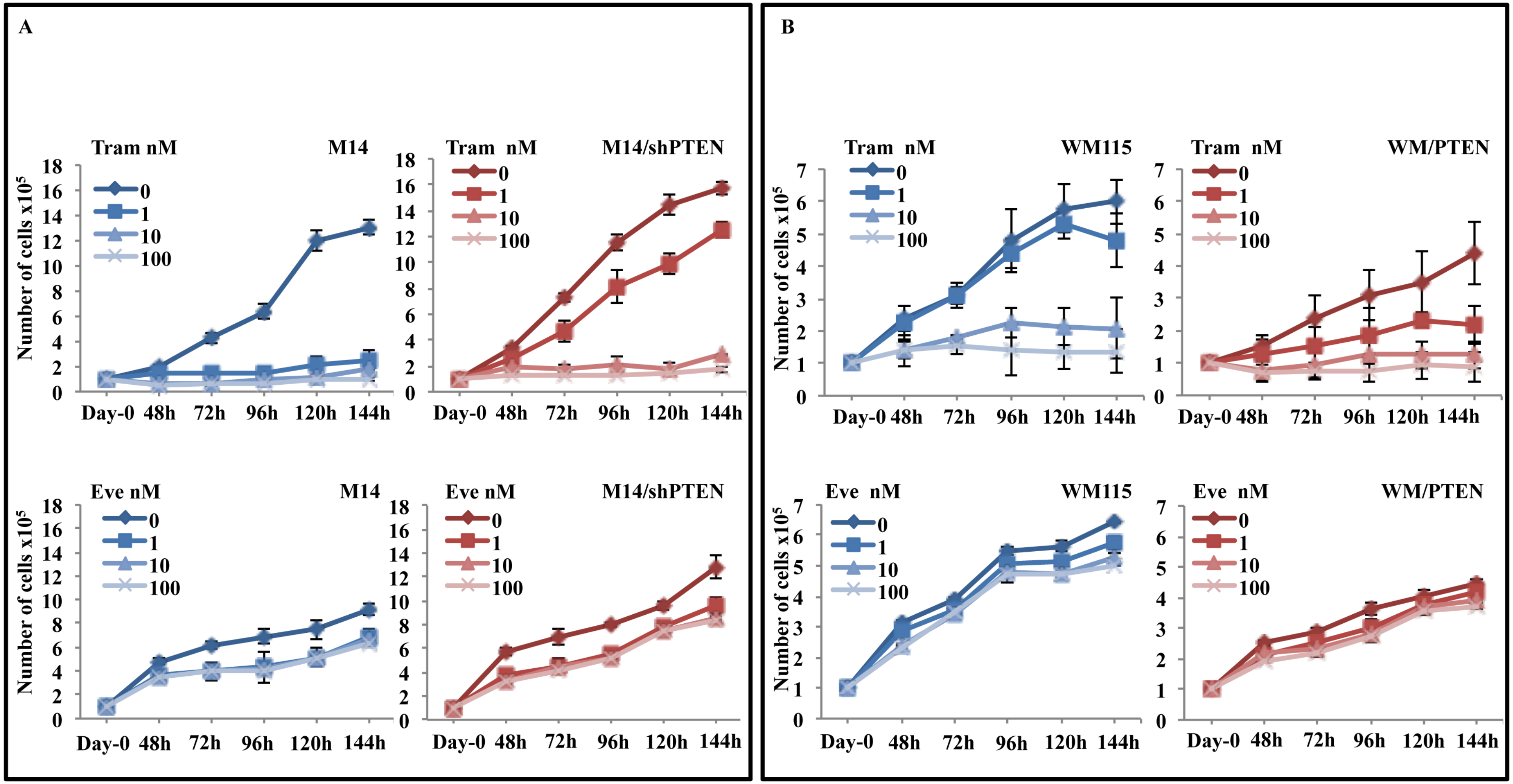
A
Tram nM
M14
0
1
10
100
Number of cells x10^5
Day-0 48h 72h 96h 120h 144h
Tram nM
M14/shPTEN
Eve nM
M14
Eve nM
M14/shPTEN
B
Tram nM
WM115
Tram nM
WM/PTEN
Eve nM
WM115
Eve nM
WM/PTEN

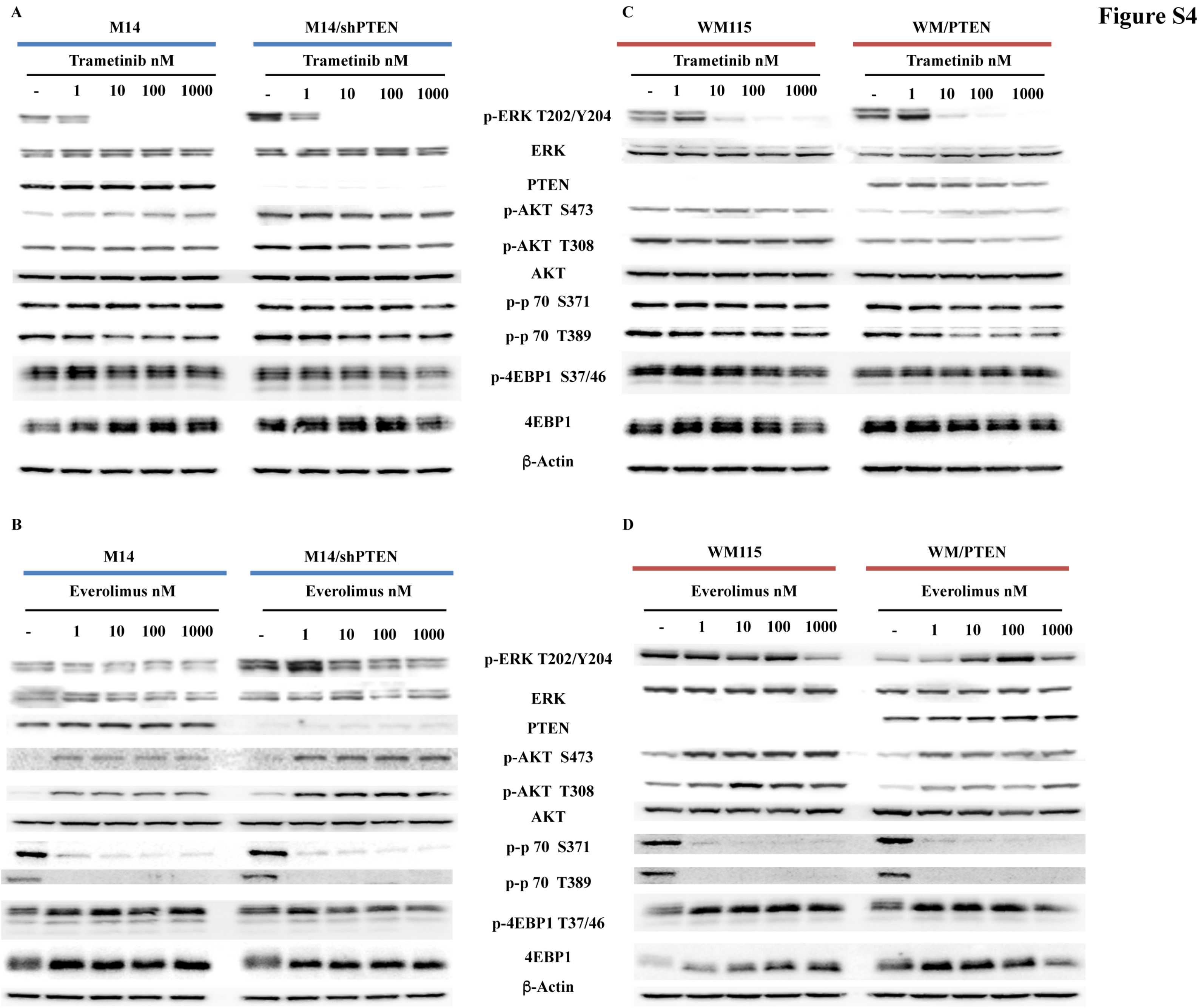
Figure S4
A
M14
M14/shPTEN
Trametinib nM
- 1 10 100 1000
C
WM115
WM/PTEN
Trametinib nM
- 1 10 100 1000
p-ERK T202/Y204
ERK
PTEN
p-AKT S473
p-AKT T308
AKT
p-p 70 S371
p-p 70 T389
p-4EBP1 S37/46
4EBP1
β-Actin
B
M14
M14/shPTEN
Everolimus nM
- 1 10 100 1000
D
WM115
WM/PTEN
Everolimus nM
- 1 10 100 1000
p-ERK T202/Y204
ERK
PTEN
p-AKT S473
p-AKT T308
AKT
p-p 70 S371
p-p 70 T389
p-4EBP1 T37/46
4EBP1
β-Actin

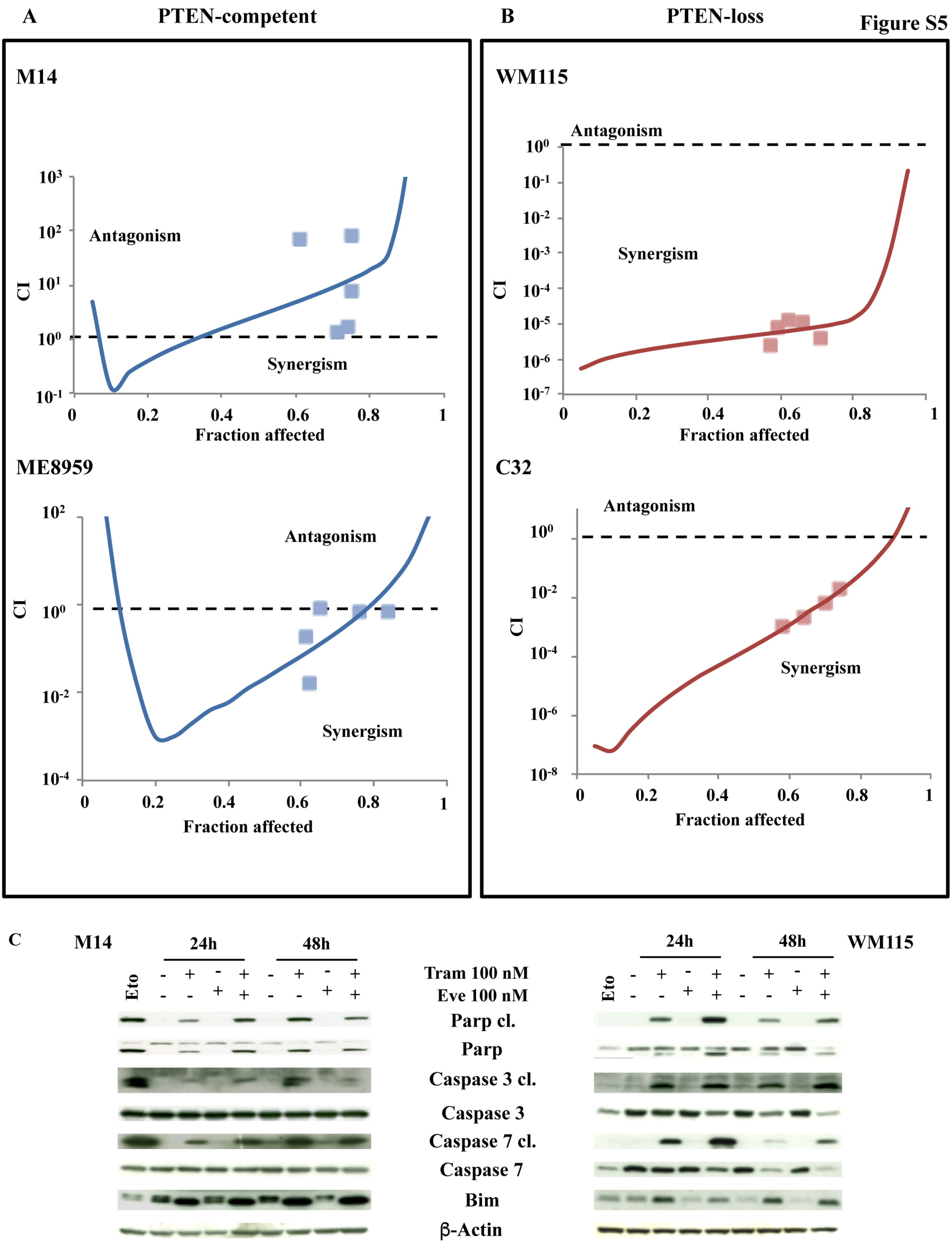
Figure S5
A
PTEN-competent
M14
CI
Antagonism
Synergism
Fraction affected
ME8959
B
PTEN-loss
WM115
C32
C
M14
24h
48h
Eto
Tram 100 nM
Eve 100 nM
Parp cl.
Parp
Caspase 3 cl.
Caspase 3
Caspase 7 cl.
Caspase 7
Bim
β-Actin
WM115

Figure S6

A

PTEN-competent

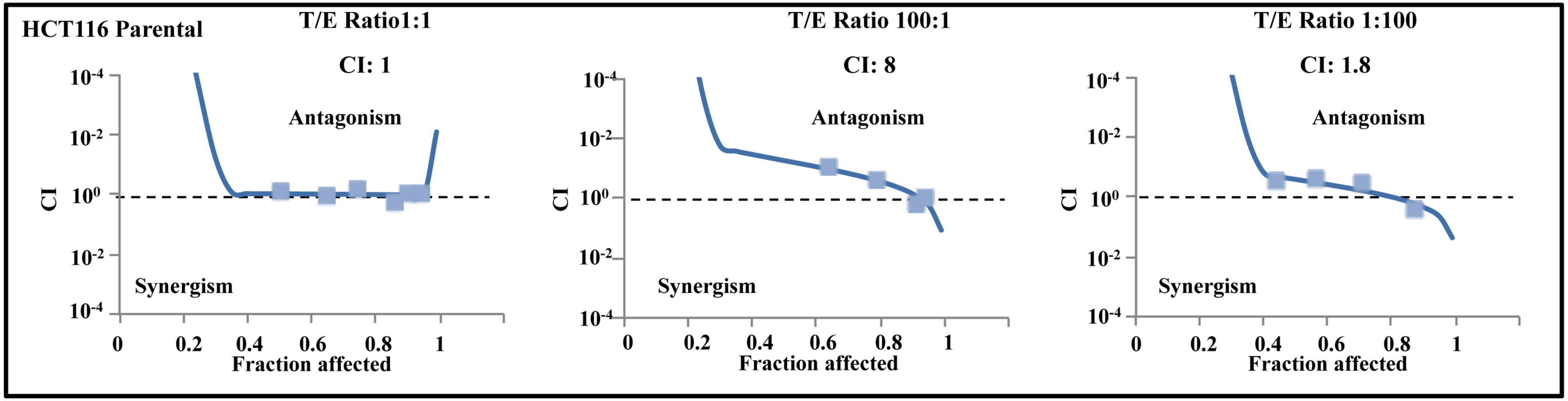

B

PTEN- loss

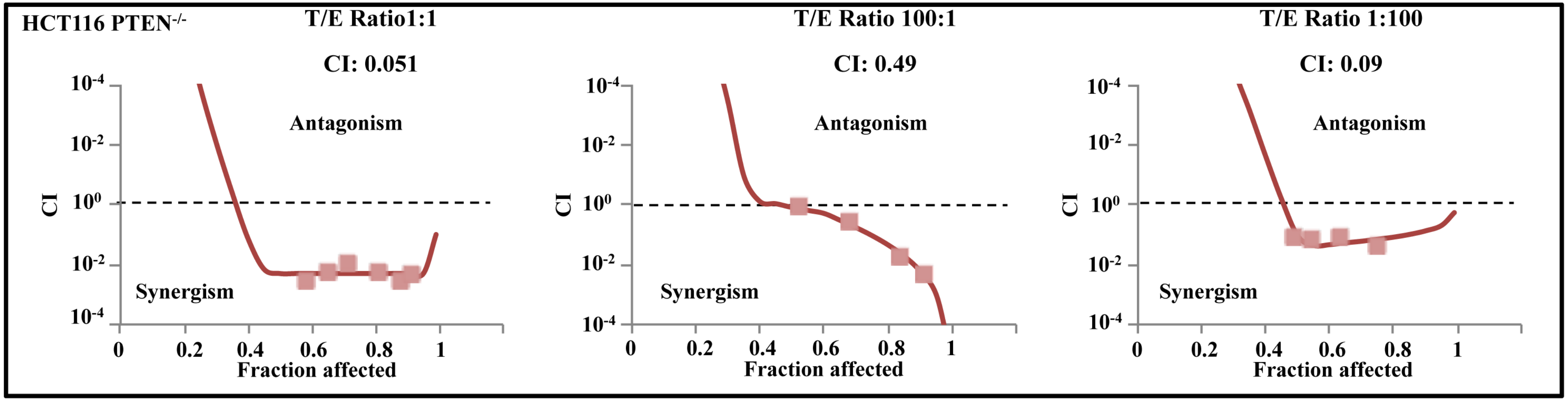

Figure S7

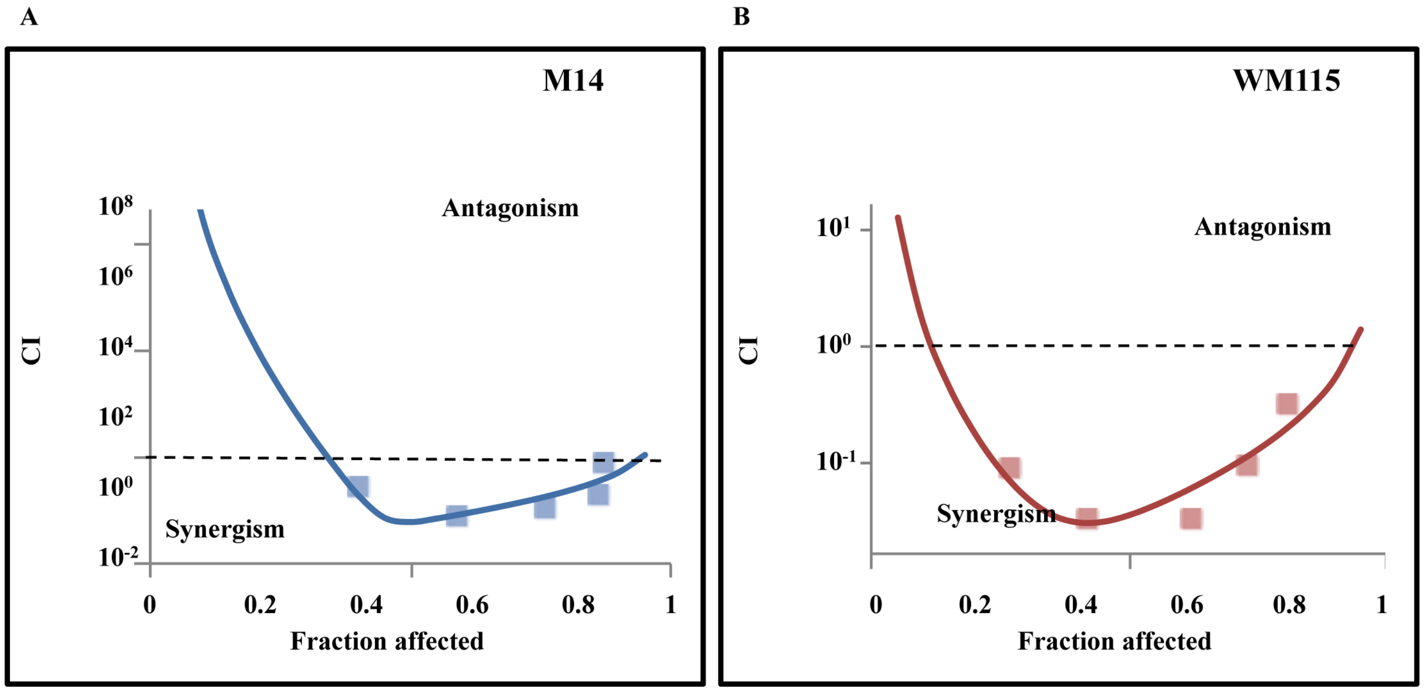
A
M14
Antagonism
Synergism
CI
10^8
10^6
10^4
10^2
10^0
10^-2
0
0.2
0.4
0.6
0.8
1
Fraction affected
B
WM115
Antagonism
Synergism
CI
10^1
10^0
10^-1
0
0.2
0.4
0.6
0.8
1
Fraction affected

Figure S8

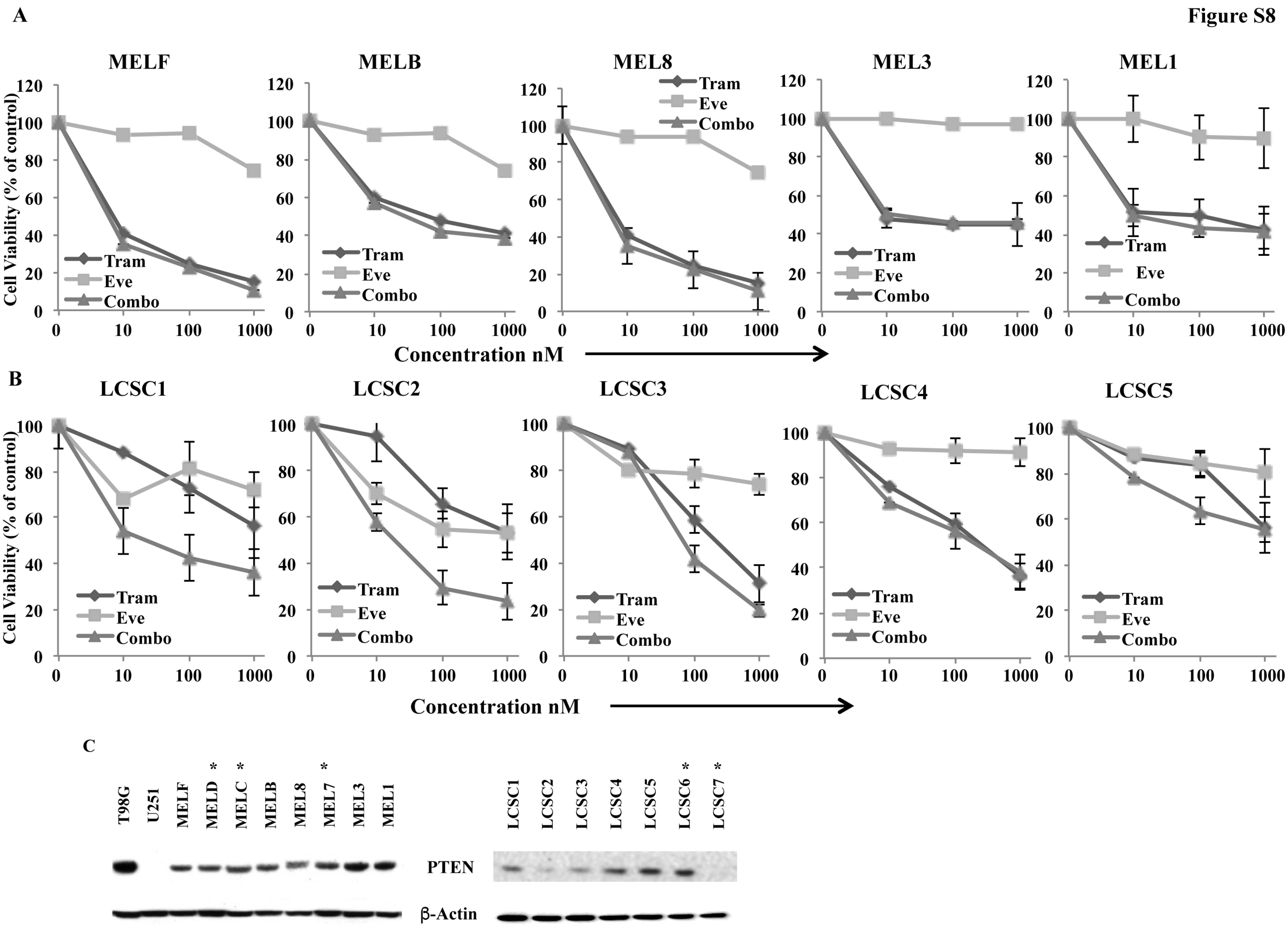

A
MELF
MELB
MEL8
MEL3
MEL1
Cell Viability (% of control)
Tram
Eve
Combo
Concentration nM
B
LCSC1
LCSC2
LCSC3
LCSC4
LCSC5
C
T98G
U251
MELF
MELD
MELC
MELB
MEL8
MEL7
MEL3
MEL1
LCSC1
LCSC2
LCSC3
LCSC4
LCSC5
LCSC6
LCSC7
PTEN
β-Actin

Figure S9

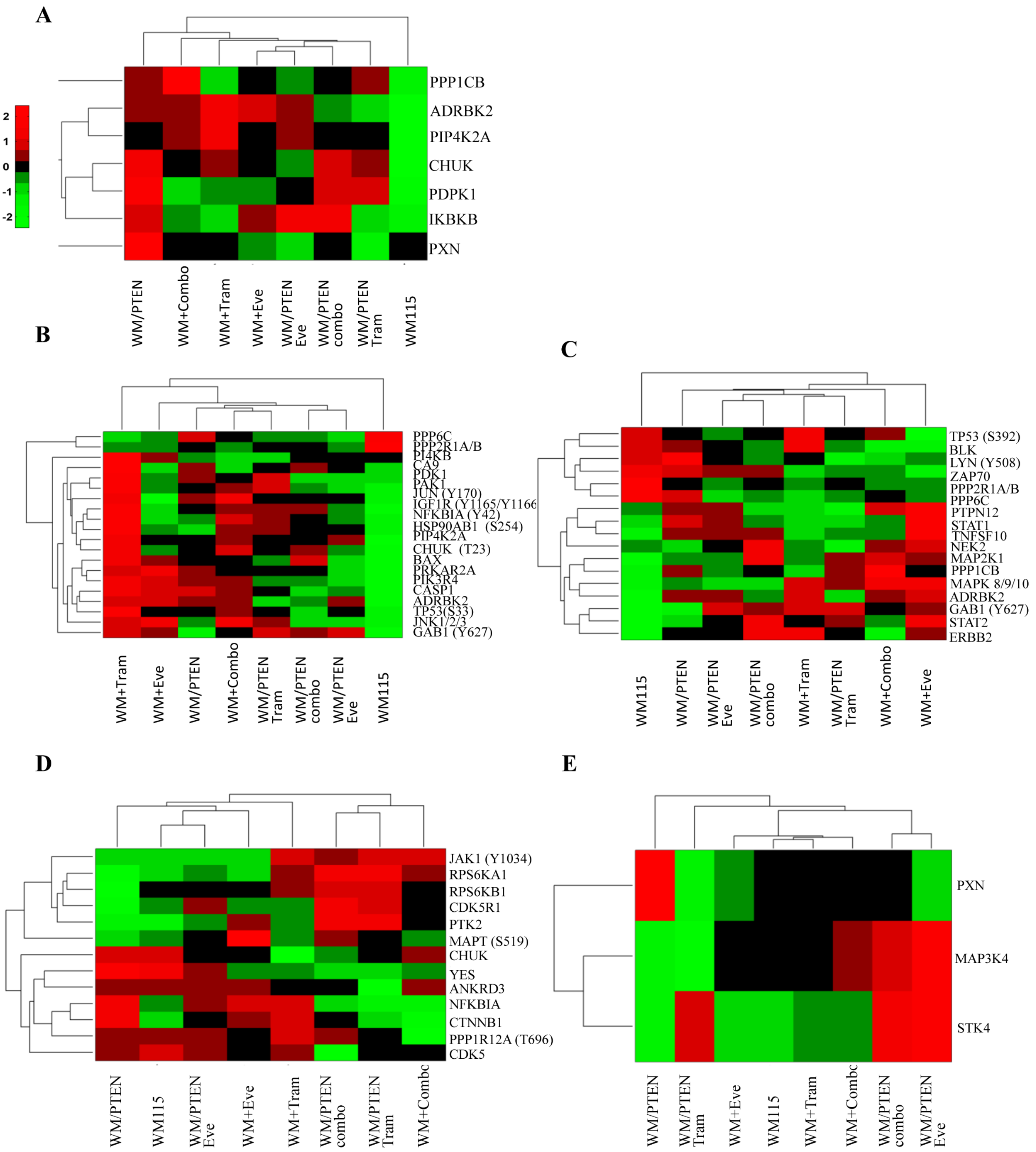
A
2
1
0
-1
-2
PPP1CB
ADRBK2
PIP4K2A
CHUK
PDPK1
IKBKB
PXN
WM/PTEN
WM+Combo
WM+Tram
WM+Eve
WM/PTEN Eve
WM/PTEN combo
WM/PTEN Tram
WM115
B
PPP6C
PPP2R1A/B
PI4KB
CA9
PDK1
PAK1
JUN (Y170)
IGF1R (Y1165/Y1166
NFKBIA (Y42)
HSP90AB1 (S254)
PIP4K2A
CHUK (T23)
BAX
PRKAR2A
PIK3R4
CASP1
ADRBK2
TP53(S33)
JNK1/2/3
GAB1 (Y627)
WM+Tram
WM+Eve
WM/PTEN
WM+Combo
WM/PTEN Tram
WM/PTEN combo
WM/PTEN Eve
WM115
C
TP53 (S392)
BLK
LYN (Y508)
ZAP70
PPP2R1A/B
PPP6C
PTPN12
STAT1
TNFSF10
NEK2
MAP2K1
PPP1CB
MAPK 8/9/10
ADRBK2
GAB1 (Y627)
STAT2
ERBB2
WM115
WM/PTEN
WM/PTEN Eve
WM/PTEN combo
WM+Tram
WM/PTEN Tram
WM+Combo
WM+Eve
D
JAK1 (Y1034)
RPS6KA1
RPS6KB1
CDK5R1
PTK2
MAPT (S519)
CHUK
YES
ANKRD3
NFKBIA
CTNNB1
PPP1R12A (T696)
CDK5
WM/PTEN
WM115
WM/PTEN Eve
WM+Eve
WM+Tram
WM/PTEN combo
WM/PTEN Tram
WM+Combo
E
PXN
MAP3K4
STK4
WM/PTEN
WM/PTEN Tram
WM+Eve
WM115
WM+Tram
WM+Combo
WM/PTEN combo
WM/PTEN Eve

Figure S10

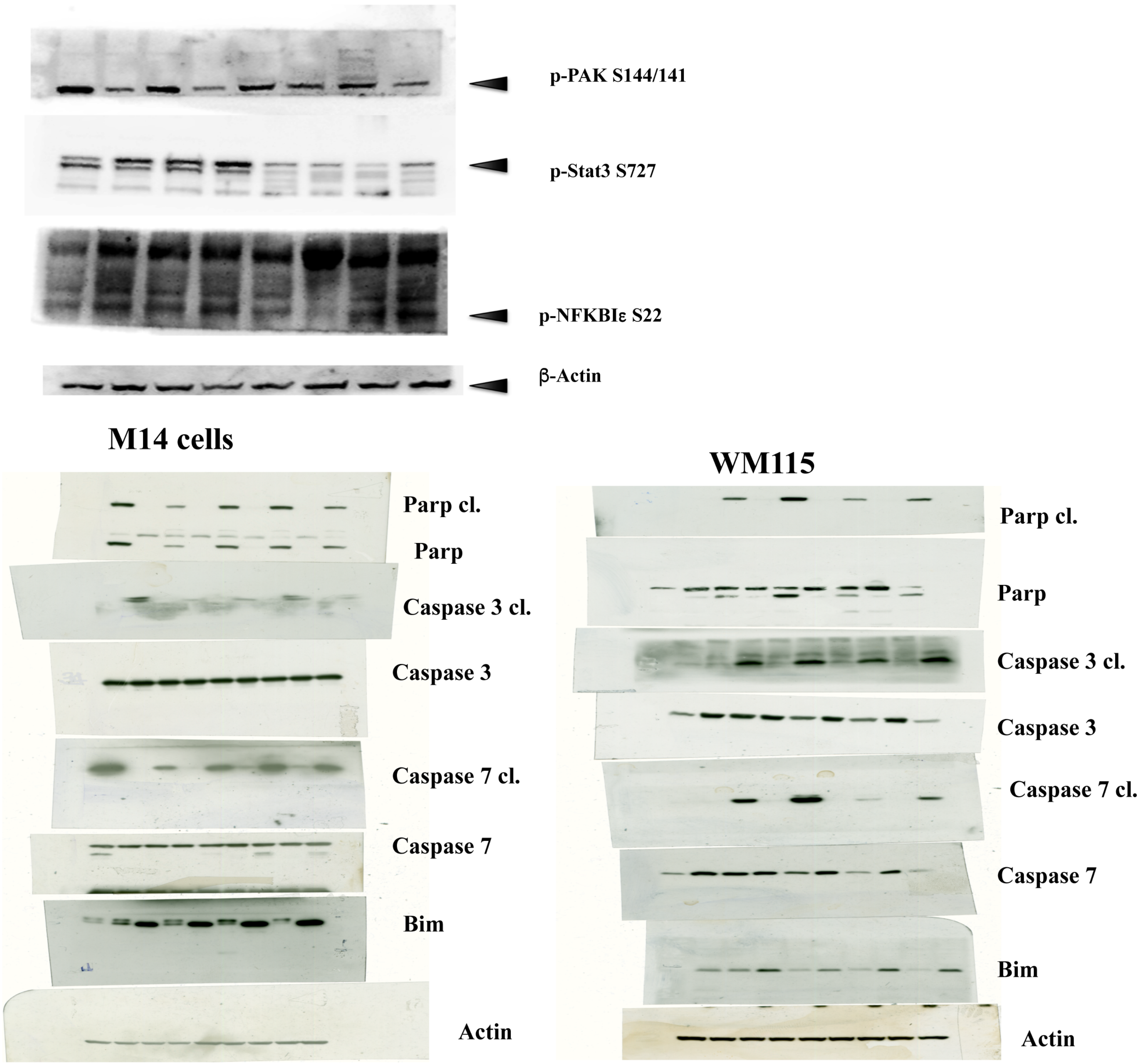
p-PAK S144/141
p-Stat3 S727
p-NFKBIε S22
β-Actin
M14 cells
Parp cl.
Parp
Caspase 3 cl.
Caspase 3
Caspase 7 cl.
Caspase 7
Bim
Actin
WM115
Parp cl.
Parp
Caspase 3 cl.
Caspase 3
Caspase 7 cl.
Caspase 7
Bim
Actin
